# Supplementary material for: Structure-guided conversion from an anaplastic lymphoma kinase inhibitor into Plasmodium lysyl-tRNA synthetase selective inhibitors
Source: Commun Biol. 2024 Jun 18;7:742. doi: 10.1038/s42003-024-06455-4 (PMC11189516; doi:10.1038/s42003-024-06455-4)
Supplement: Supplementary file 2 — Supplementary Information 20240608 [file 42003_2024_6455_MOESM2_ESM.pdf]

Supplementary Information for

Structure-guided conversion from an anaplastic lymphoma kinase inhibitor into *Plasmodium* lysyl-tRNA synthetase selective inhibitors

Jintong Zhou[1,2,†], Mingyu Xia[2,†], Zhenghui Huang[3], Hang Qiao[2], Guang Yang[1], Yunan Qian[3], Peifeng Li[2], Zhaolun Zhang[4], Xinai Gao[4], Lubin Jiang[3], Jing Wang[1,2,*], Wei Li[4,*], Pengfei Fang[1,2,*]

[1]School of Chemistry and Materials Science, Hangzhou Institute for Advanced Study, University of Chinese Academy of Sciences, 1 Sub-lane Xiangshan, Hangzhou 310024, China.

[2]State Key Laboratory of Chemical Biology, Shanghai Institute of Organic Chemistry, University of Chinese Academy of Sciences, Chinese Academy of Sciences, 345 Lingling Road, Shanghai 200032, China.

[3]Key Laboratory of Molecular Virology and Immunology, Shanghai Institute of Immunity and Infection, Chinese Academy of Sciences, 320 Yueyang Road, Shanghai 200031, China.

[4]Department of Medicinal Chemistry, School of Pharmacy, China Pharmaceutical University, 639 Longmian Avenue, Nanjing, Jiangsu 211198, China.

[†]These authors contributed equally to this work: J.Z., M.X.

[*]To whom correspondences should be addressed. Emails:

jwang@sioc.ac.cn (J.W.), wli@cpu.edu.cn (W.L.), or fangpengfei@sioc.ac.cn (P.F.).

Contents

Supplementary Figures 3

Supplementary Figure 1 3

Supplementary Figure 2 4

Supplementary Figure 3 5

Supplementary Figure 4 6

Supplementary Figure 5 7

Supplementary Figure 6 8

Supplementary Figure 7 9

Supplementary Figure 8 10

Supplementary Figure 9 11

Supplementary Figure 10 12

Supplementary Figure 11 13

Supplementary Figure 12 14

Supplementary Figure 13 15

Supplementary Tables 16

Supplementary Table 1 16

Supplementary Table 2 18

Supplementary Table 3 19

Supplementary Table 4 20

Supplementary Table 5 21

Supplementary Note 1 22

Supplementary Figures

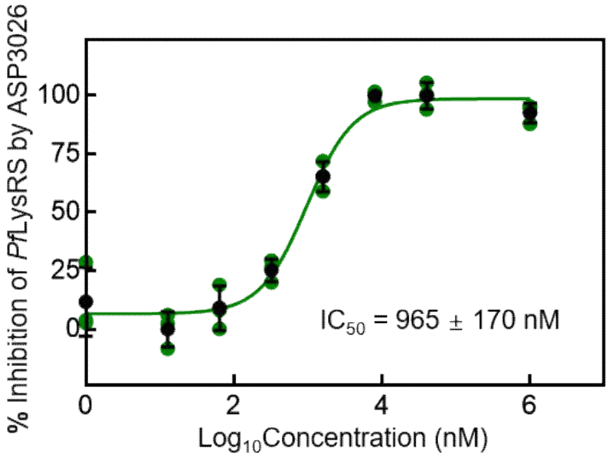

Supplementary Figure 1. The potency of compound ASP3026 against *Pf*LysRS is measured using the ATP hydrolysis assay. Error bars represent SD of three technical repeats.

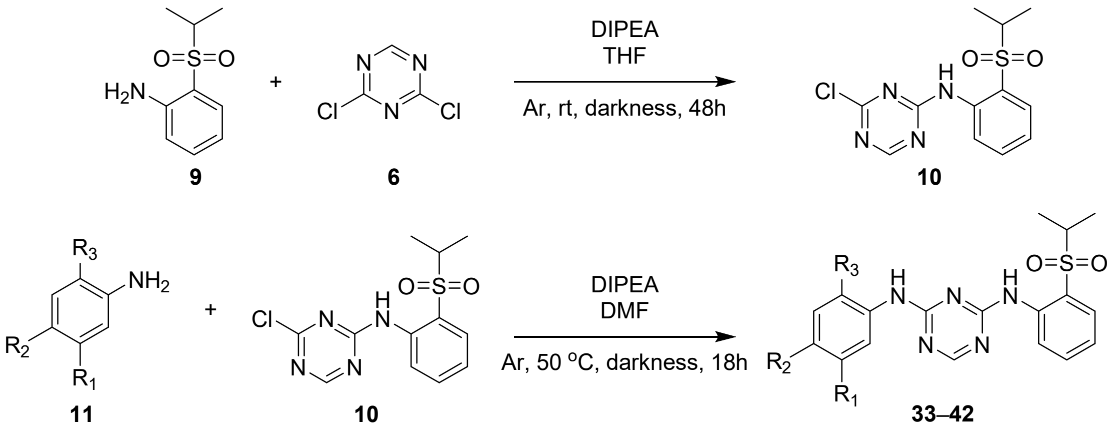

Supplementary Figure 2. Synthesis of compounds 33–42. The detailed methods for synthesis of the compounds are included in the Supplementary Note 1.

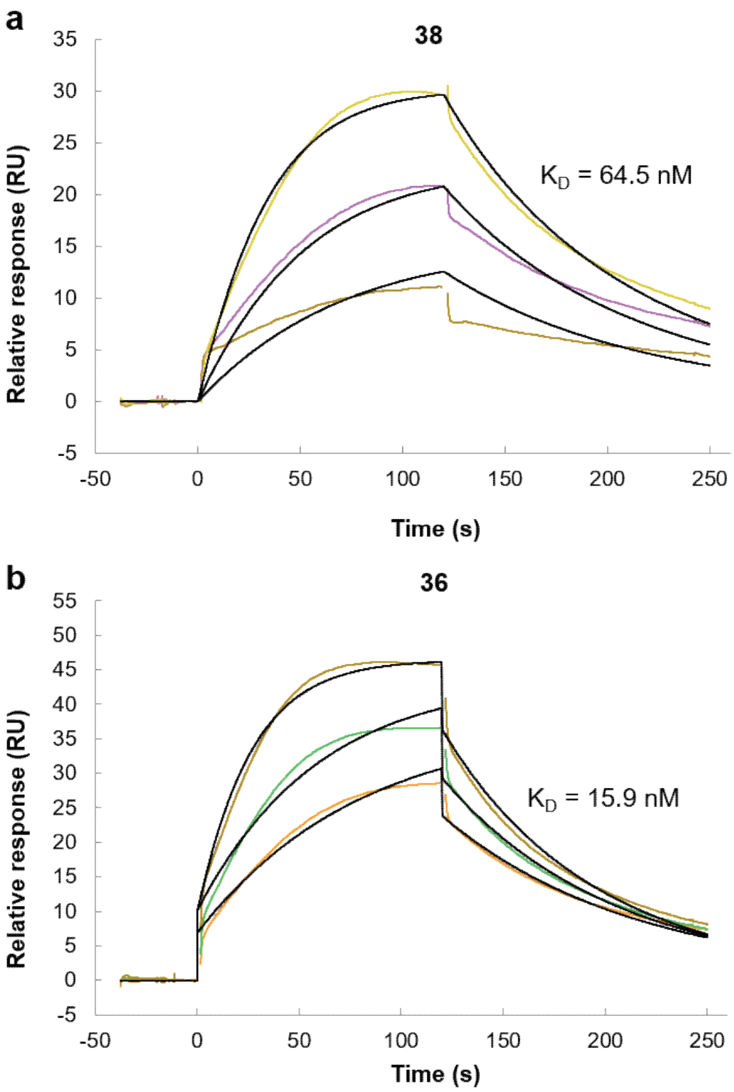

Supplementary Figure 3. Surface plasmon resonance (SPR) sensorgrams for the interaction of compounds 38 and 36 with immobilized *Pf*LysRS. (a) The colored curves represent measurements of different concentrations of compound **38**. From top to bottom, they are 125 nM, 62.5 nM, and 31.2 nM, respectively. The black curves represent the corresponding fit curves. k_{on}=2.18E+05 $(M\cdot s)^{-1}$, k_{off}=1.37E-02 s^{-1}, and K_D=62.8 nM. (**b**) The colored curves represent actual measurements of different concentrations of compound **36**. From top to bottom, they are 31.2 nM, 7.8 nM, and 3.9 nM, respectively. The black curves represent the corresponding fit curves. k_{on}=9.07E+05 $(M\cdot s)^{-1}$, k_{off}=1.44E-02 s^{-1}, and K_D=15.9 nM.

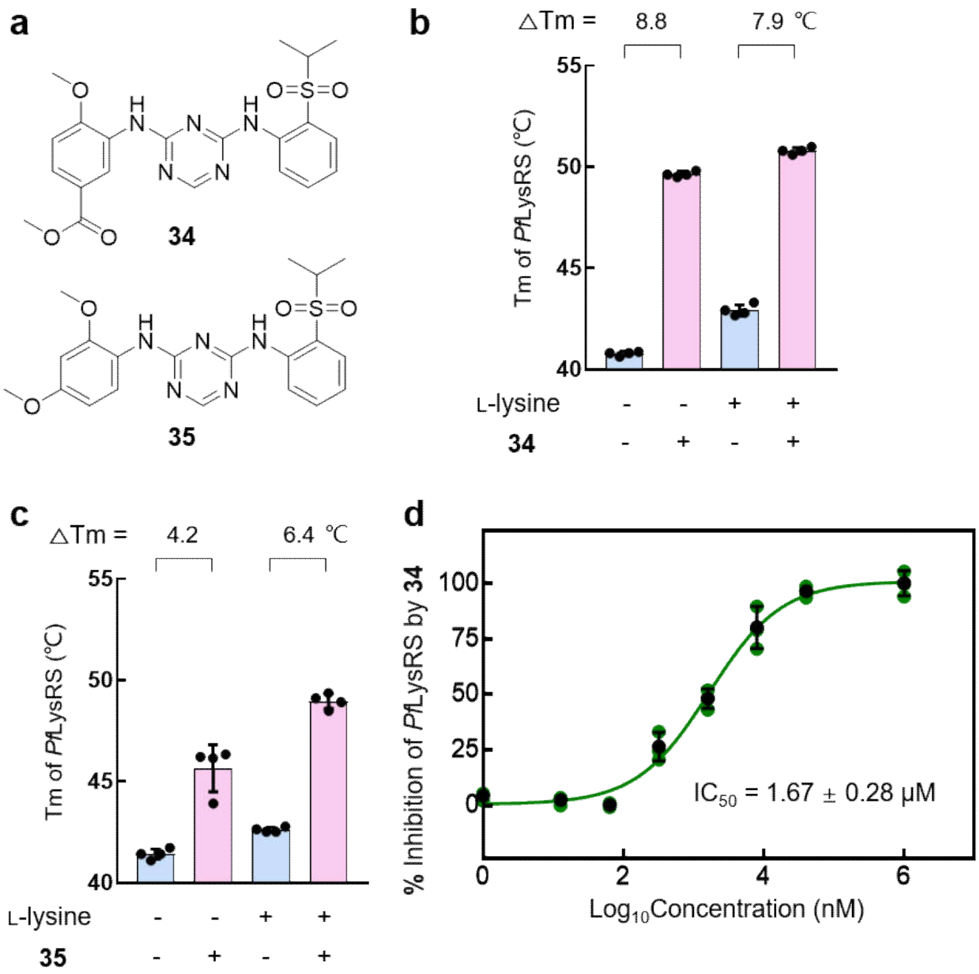

Supplementary Figure 4. Chemical structures and activities of compound 34 and 35. (**a**) Chemical structures of compound **34** and **35**. (**b**) Diagram of the Tms of *Pf*LysRS in the presence of L-lysine and/or compound **34**. (**c**) Diagram of the Tms of *Pf*LysRS in the presence of L-lysine and/or compound **35**. Error bars in **b-c** represent SD of four technical repeats. (**d**) The potency of compound **34** against *Pf*LysRS is measured using the ATP hydrolysis assay. Error bars represent SD of three technical repeats.

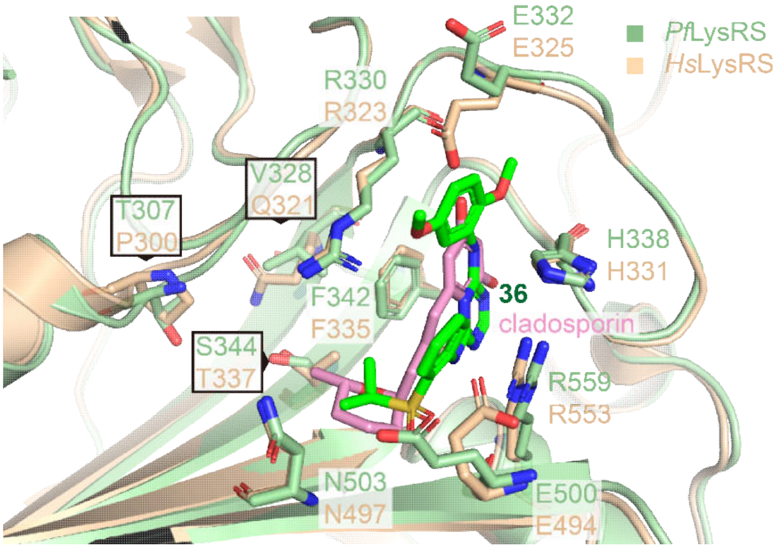

Supplementary Figure 5. The superposition of *Pf*LysRS and *Hs*LysRS structural cavities. *Pf*LysRS (PDB code: 8K9V) was shown as light green cartoons and sticks. *Hs*LysRS (PDB code: 4YCU) was shown as wheat cartoons and sticks. The compound **36** (green sticks) occupies a space in the ATP binding pocket of LysRS and overlap mostly with cladosporin (pink sticks). Most of the compound binding residues are conserved between *Pf*LysRS and *Hs*LysRS. Three different residues (Thr307/Pro300, Val328/Gln321, and Ser344/Thr337) are indicated with black boxes.

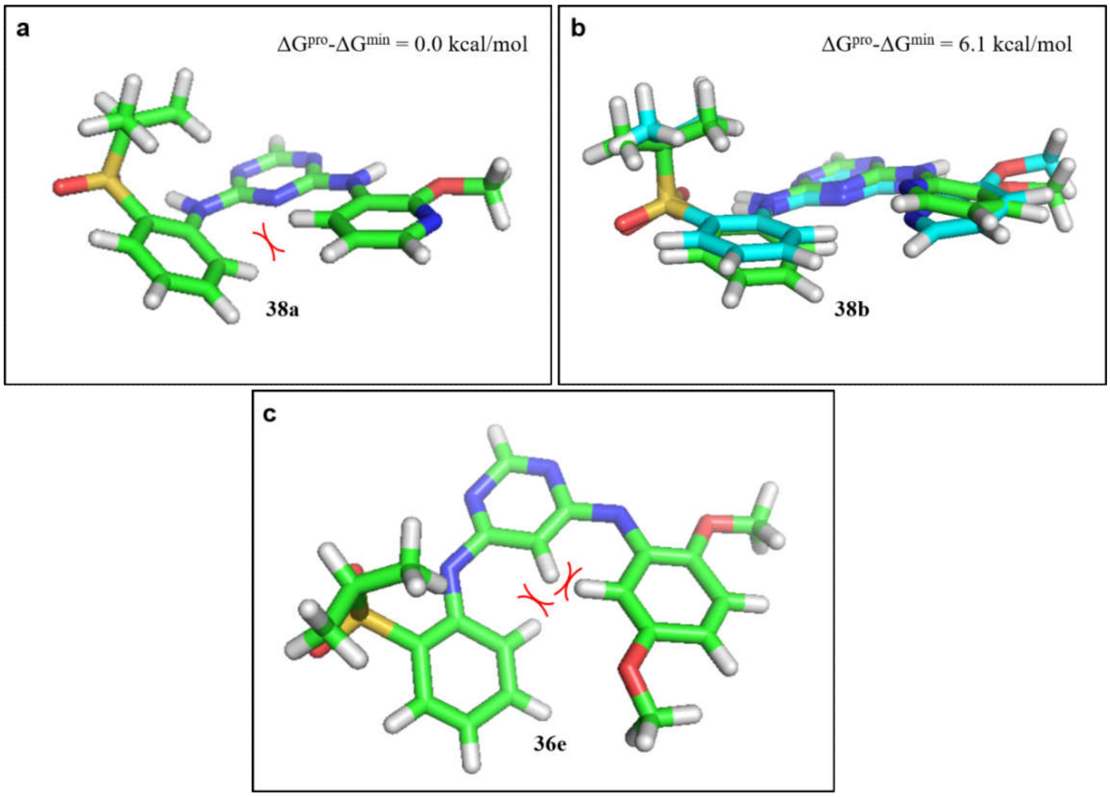

Supplementary Figure 6. Effect of aromatic ring substitutions on the conformation of compounds. (**a**) The superposition of the minimum energy conformation of compound **38a** and the *Pf*LysRS protein binding conformation. (**b**) The superposition of the minimum energy conformation of compound **38b** and the protein binding conformation. Green represents the *Pf*LysRS protein binding conformation. Cyan represents the minimum local energy conformation. (**c**) The *Pf*LysRS protein binding conformation of **36e** refer to the conformation of compound **38** in the crystal structure *Pf*LysRS-**38**. DFT calculations at the quantum chemical level B3LYP-D3(BJ)/6-31G** for structure optimization and frequency, with single-point energy correction at the higher B3LYP-D3(BJ)/6-311+G** level.

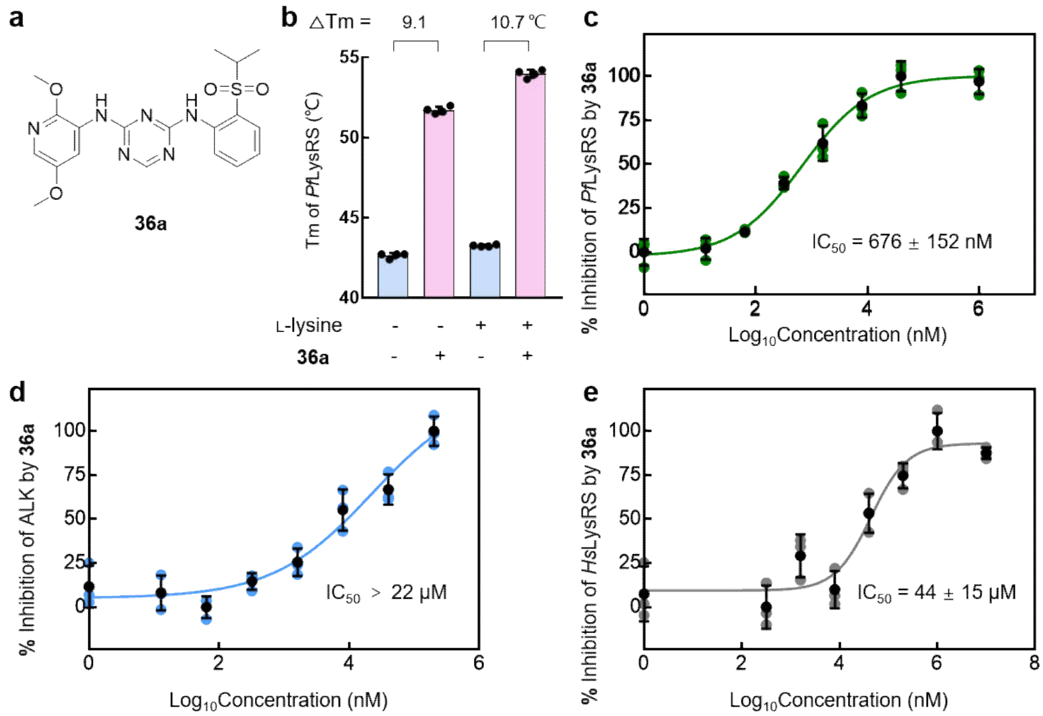

Supplementary Figure 7. The replacement of methoxy-linked benzene ring with pyridine only enhances the inhibition ability against *Hs*LysRS. (**a**) Chemical structure of compound **36a**. (**b**) Diagram of the Tms of *Pf*LysRS in the presence of L-lysine and/or compound **36a**. Error bars represent standard deviations (SD) of four technical repeats. (**c**) The potency of compound **36a** against *Pf*LysRS is measured using the ATP hydrolysis assay. (**d**) The potency of compound **36a** against ALK is measured using the ATP hydrolysis assay. (**e**) The potency of compound **36a** against *Hs*LysRS is measured using the ATP hydrolysis assay. Error bars in **c-e** represent SD of three technical repeats.

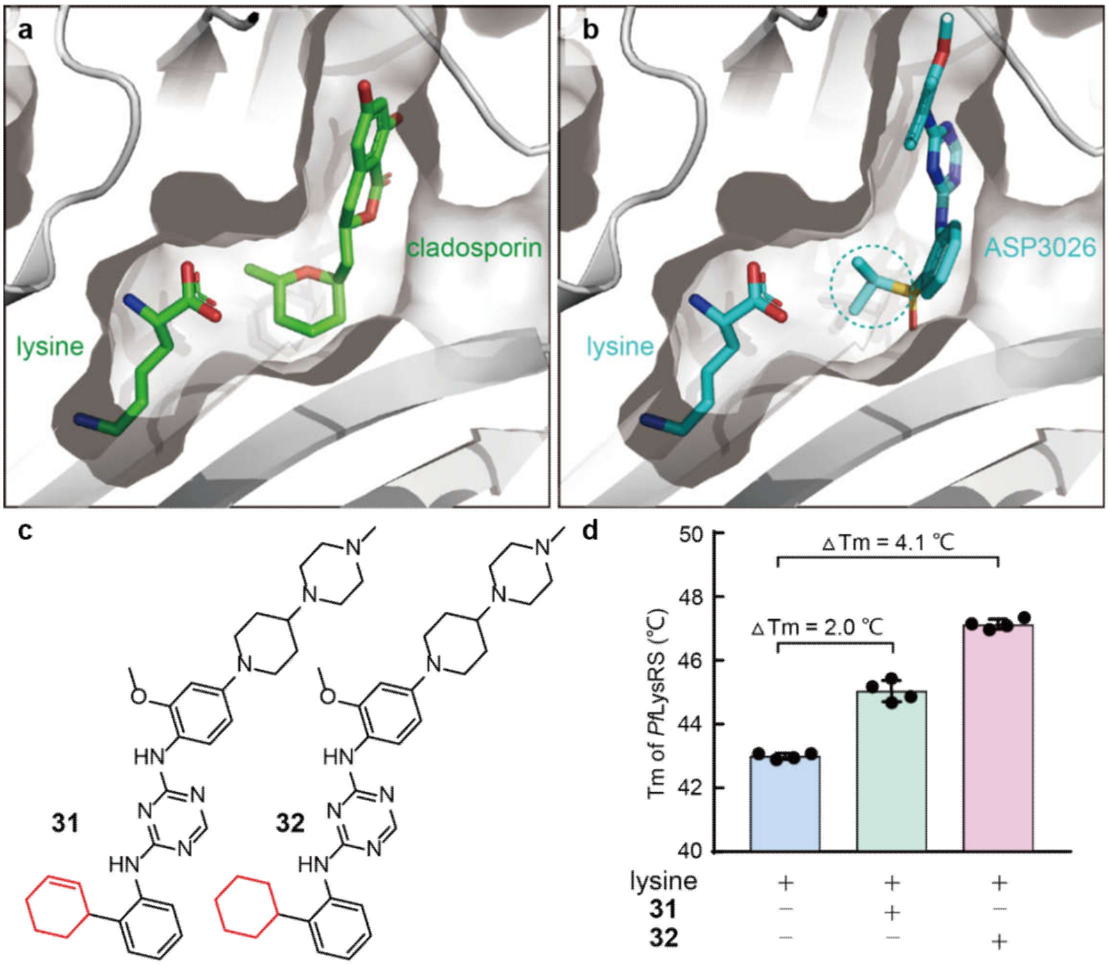

Supplementary Figure 8. Substitution of isopropylsulfonyl moiety was designed to create synergistic binding. (**a**) The van der Waals interaction between the cladosporin and L-lysine enhances their synergistic effect (PDB code: 4YCV). Cladosporin and L-lysine are depicted as sticks. (**b**) Replacing the isopropyl sulfonyl part of ASP3026 with a larger moiety to mimic the van der Waals interaction between cladosporin and L-lysine may enhance the synergistic effect. ASP3026 and L-lysine are depicted as sticks. (**c**) Chemical structures of compound **31** and **32**. (**d**) Diagram of the Tms of *Pf*LysRS in the presence of L-lysine and compound **31** or **32**. Error bars represent standard deviations (SD) of four technical repeats.

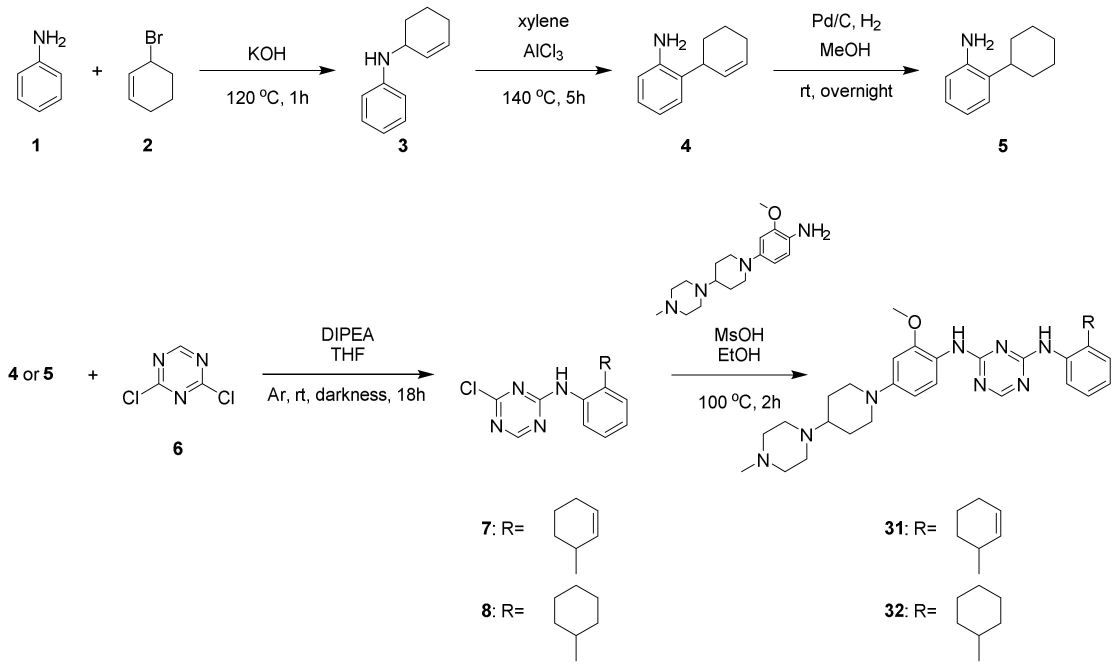

Supplementary Figure 9. Synthesis of compounds 31 and 32. The detailed methods for synthesis of the compounds are included in the Supplementary Note 1.

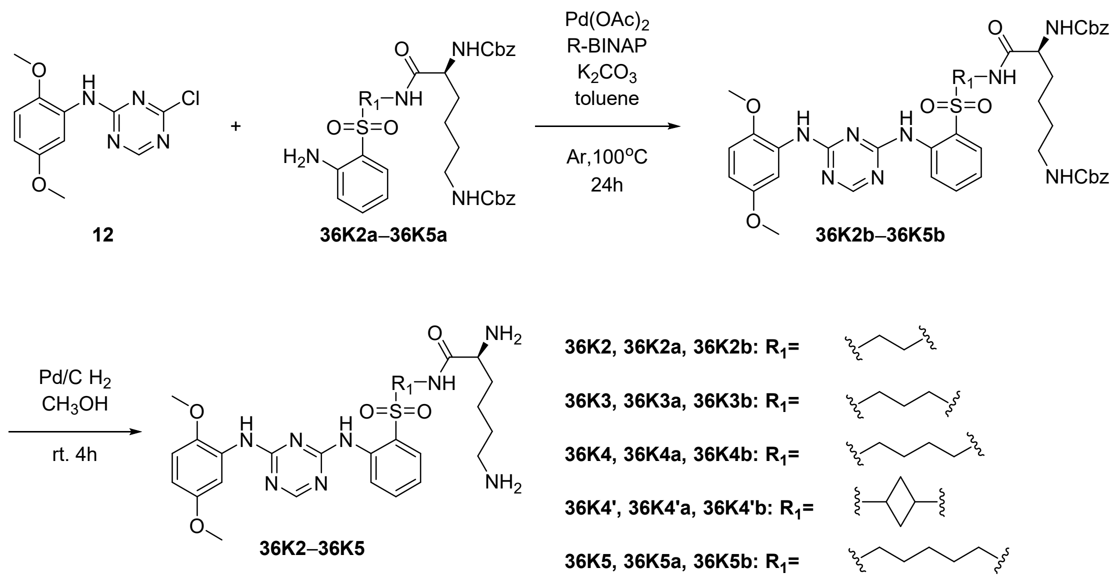

Supplementary Figure 10. Synthesis of compounds 36K2–36K5. The detailed methods for synthesis of the compounds are included in the Supplementary Note 1.

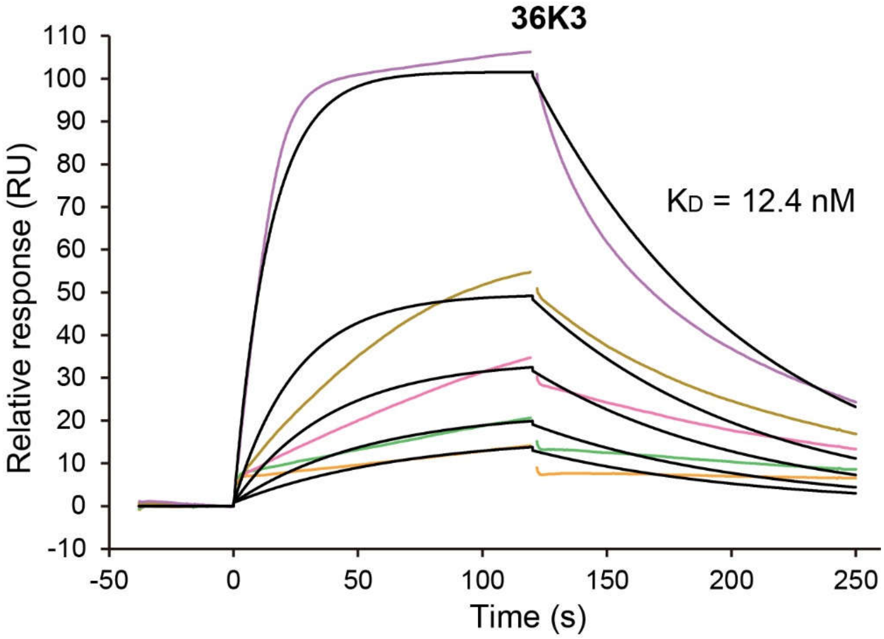

Supplementary Figure 11. Surface plasmon resonance (SPR) sensorgrams for the interaction of 36K3 with immobilized *Pf*LysRS. The colored curves represent measurements of different concentrations of compound **36K3**. From top to bottom, they are 62.5 nM, 31.2 nM, 15.6 nM, 7.8 nM, and 3.9 nM, respectively. The black curves represent the corresponding fit curves. k_{on}=9.11E+05 (M·s)$^{-1}$, k_{off}=1.13E-02 s^{-1}, and K_D=12.4 nM.

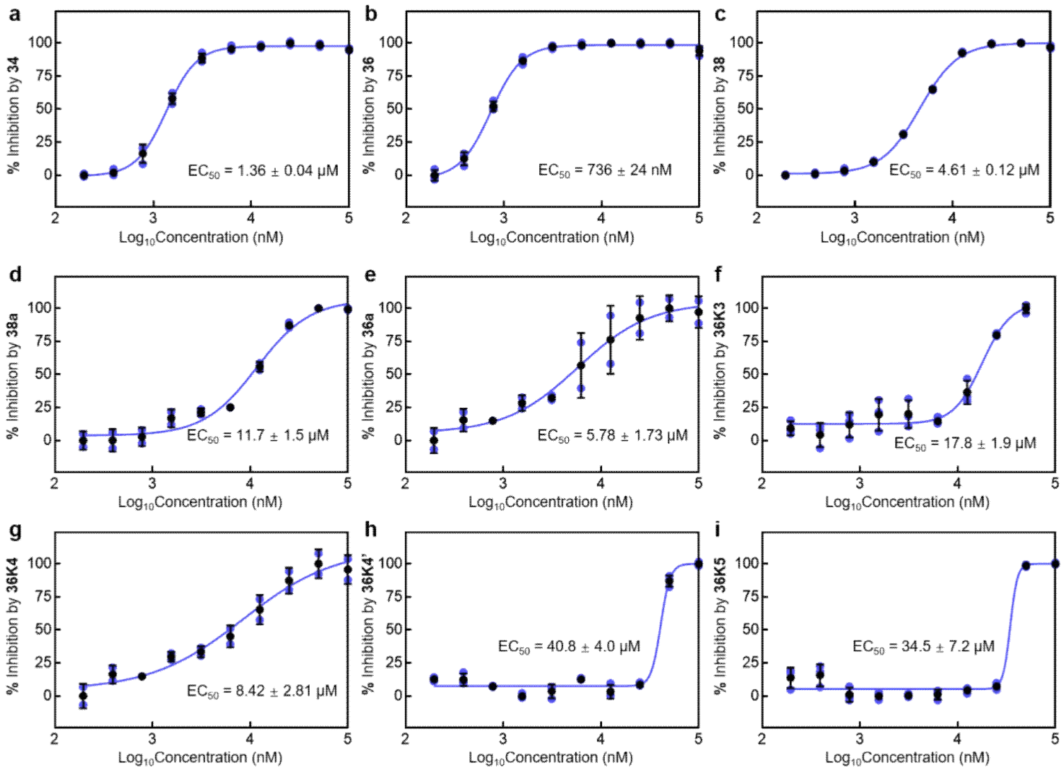

Supplementary Figure 12. ASP3026 analogues can inhibit the growth of erythrocytic-stage *P. falciparum* 3D7 parasites. (**a-i**) The potencies of compounds **34**, **36**, **38**, **38a**, **36a**, **36K3**, **36K4**, **36K4'** and **36K5** against the growth of erythrocytic-stage *P. falciparum* 3D7 parasites. Error bars in **a-i** represent SD of two or three technical repeats.

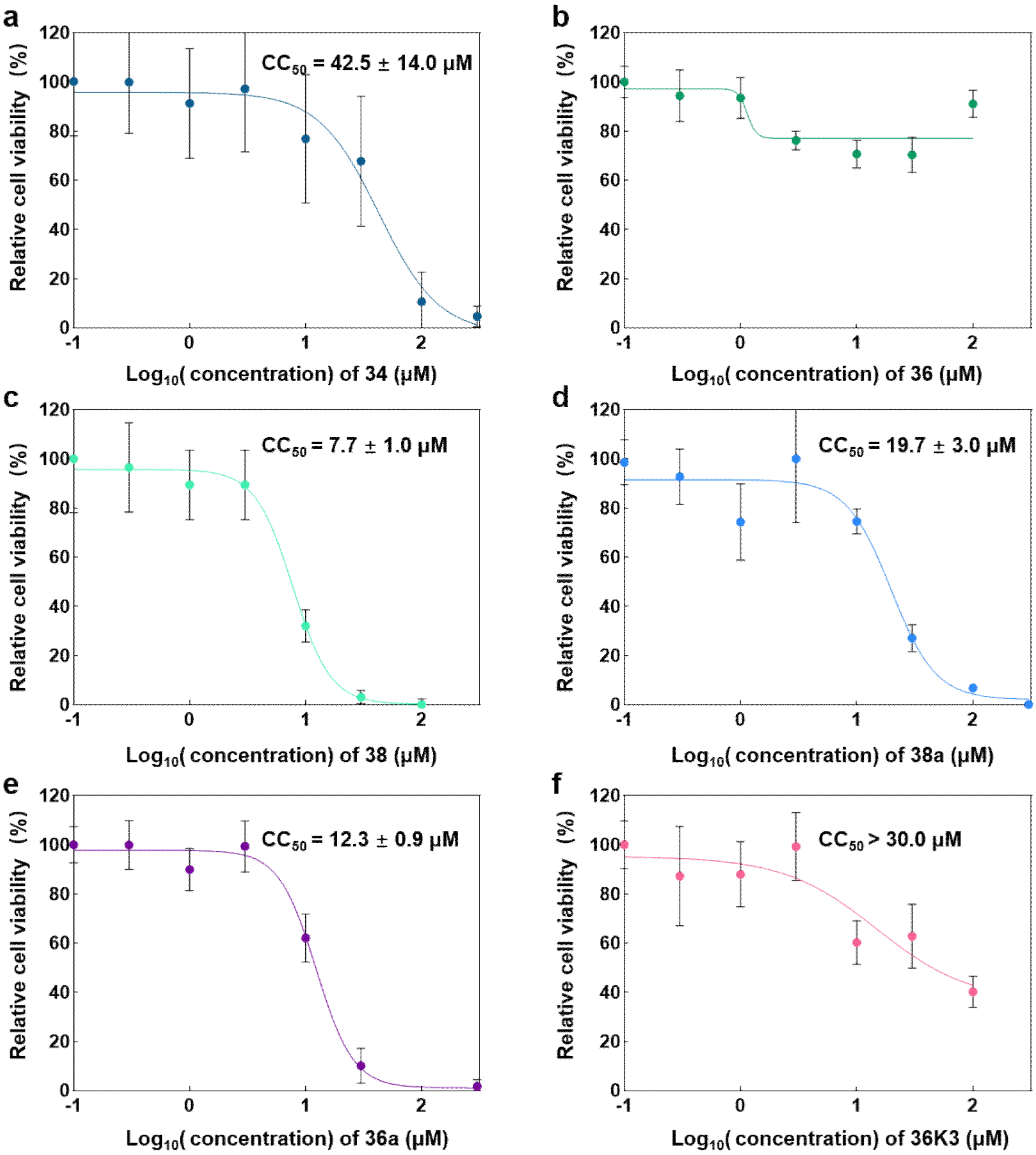

Supplementary Figure 13. Cytotoxicity of some analogues against HepG2 cell line. (a-f) The cytotoxicity of compounds **34**, **36**, **38**, **38a**, **36a** and **36K3** against the growth of HepG2 cell line. Error bars in **a-f** represent SD of three technical repeats.

Supplementary Tables

Supplementary Table 1. Data collection and refinement statistics

	*Pf*LysRS-**34**	*Pf*LysRS-**35**
**PDB code**	8K9S	8K9U
**Data collection**		
Space group	$P2_1\,2_1\,2_1$	$P2_1\,2_1\,2_1$
cell dimensions		
a, b, c (Å)	71.46, 95.89, 167.50	69.72, 92.26, 162.62
α, β, γ (°)	90, 90, 90	90, 90, 90
Resolution (Å)	48.25-2.35 (2.43-2.35)	46.74-2.83 (2.98-2.83)
R_{sym} or R_{merge} (%)	19.9(110.0)	20.8(147.8)
I/σ(I)	9.1(2.1)	11.9(1.9)
Completeness (%)	99.8(100.0)	99.9(100.0)
Redundancy	6.3(6.7)	13.2(13.4)
**Refinement**		
Resolution (Å)	48.25-2.35 (2.43-2.35)	46.74-2.83 (2.94-2.83)
Total reflections	48610	25715
R_{work} / R_{free} (%)	21.9 / 26.3	22.5 / 28.3
No. atoms		
Protein	7714	7551
Ligand	84	80
Solvent	317	108
B-factors		
Protein	56.55	71.01
Ligand	53.06	59.22
Solvent	54.51	54.00
R.m.s. deviations		
Bond length (Å)	0.003	0.004
Bond angle (°)	0.633	0.731
Ramachandran plot		
Most favored [%]	97.91	97.78
Additional allowed [%]	2.09	2.22

Statistics for the highest-resolution shell are shown in parentheses.

Continuation of Supplementary Table 1

*Pf*LysRS-**36**	*Pf*LysRS-**38**	*Pf*LysRS-**36K3**
8K9V	8K9W	8K9X
$P2_1 2_1 2_1$	$P2_1 2_1 2_1$	$P2_1 2_1 2_1$
71.15, 99.13, 169.01	71.92, 95.55, 168.25	71.67, 99.49, 171.42
90, 90, 90	90, 90, 90	90, 90, 90
48.98-1.92 (1.95-1.92)	48.37-2.30 (2.37-2.30)	48.12-2.35 (2.42-2.35)
9.2(112.4)	7.5(112.8)	14.3(123.9)
11.0(1.6)	13.8(2.1)	10.1(1.9)
100.0(100.0)	100.0(100.0)	99.6(100.0)
6.4(5.0)	7.3(7.5)	7.1(7.7)
47.56-1.92 (1.99-1.92)	32.45-2.30 (2.38-2.30)	48.12-2.35 (2.41-2.35)
91847	52243	51432
18.7 / 22.0	21.4 / 25.1	24.9 / 27.6
7778	7511	7667
80	56	80
476	160	130
45.99	73.98	56.60
35.18	69.62	52.34
48.90	65.85	53.99
0.008	0.006	0.011
1.005	0.847	1.535
97.51	97.23	97.68
2.49	2.77	2.32

Supplementary Table 2. Structures and ΔTms of compounds **33–42.**

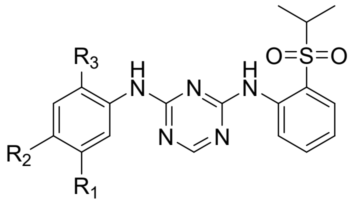

Compound	R_1	R_2	R_3	ΔTm (°C)
**ASP3026**				8.9
**33**	—CF_3	—H	—O/	-0.1
**34**	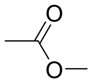	—H	—O/	7.9
**35**	—H	—O/	—O/	6.4
**36**	—O/	—H	—O/	11.8
**37**	—H	—H	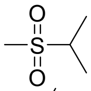	0.5
**38**	—H	—H	—O/	9.0
**39**	—CH_3	—H	—O/	4.1
**40**	—NO_2	—H	—O/	2.5
**41**	—F	—H	—O/	1.9
**42**	—OCF_3	—H	—O/	1.5

Supplementary Table 3. Structures and ΔTms of compounds **36**, **38**, **38a**, **38b** and **36a**

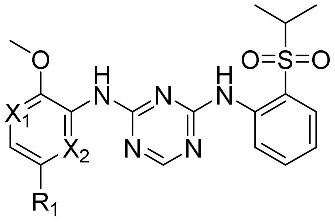

Compound	X_1	X_2	R_1	ΔTm (^{o}C)
**ASP3026**				8.9
**36**	C	C	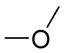	11.8
**38**	C	C	—H	9.0
**38a**	N	C	—H	7.9
**38b**	C	N	—H	2.0
**36a**	N	C	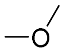	10.7

Supplementary Table 4. Structures and ΔTms of compounds **36**, **36b–f**

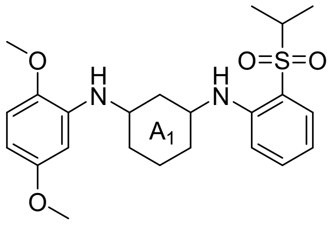

Compound	A_1	ΔTm (°C)
**ASP3026**		8.9
**36**	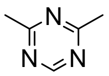	11.8
**36b**	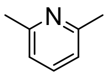	0.1
**36c**	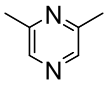	0
**36d**	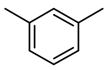	−0.2
**36e**	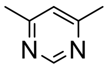	3.1
**36f**	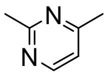	2.4

Supplementary Table 5. Enzyme inhibitory activities of compounds **36K2–36K5**

IC_{50}	**36K2**	**36K3**	**36K4**	**36K4'**	**36K5**
ALK (μM)	>109	>74.4	>79.0	>170	>66.8
*Pf*LysRS (nM)	365	59.2	377	298	2770
*Hs*LysRS (μM)	31	12	11	26	22

Supplementary Note 1

Methods of Compound Synthesis

All reactions were carried out under an argon atmosphere with dry solvents under anhydrous conditions, unless otherwise noted. Tetrahydrofuran (THF) was distilled immediately before use from sodium. Dichloromethane (CH_2Cl_2), N,N-dimethylformamide (DMF) and toluene were dried with activated Linde types 4Å molecular sieves and stored under an argon atmosphere. Methanol (MeOH) was dried with activated Linde types 3Å molecular sieves and stored under an argon atmosphere. All the commercially available chemicals were purchased from local suppliers and used as received unless otherwise noted. Solvents for chromatography were used as supplied by Adamas-beta®. Thin-layer chromatography was performed using Merck Si60 F254 precoated glass plates and visualized by CAM or 3-methoxyphenol/sulfuric acid staining. Huanghai silica gel (particle size 200–300 mesh) was used for flash chromatography. ^{1}H and ^{13}C NMR spectra were recorded on a Bruker BioSpin GmbH 300 MHz, a Bruker Avance III 400 MHz, an Agilent DD2 500 MHz, or a Bruker Avance III HD 600 MHz spectrometer at room temperature. The spectra were calibrated by using residual undeuterated solvents (for ^{1}H NMR) and deuterated solvents (for ^{13}C NMR) as internal references: $CHCl_3$ (δH = 7.26 ppm) and $CDCl_3$ (δC = 77.16 ppm); CHD_2OD (δH = 3.31 ppm) and methanol-d_4 (δC = 49.00 ppm); pyridine-d_5 (δH = 7.20, 7.57, 8.72 ppm) and pyridine-d_5 (δC = 123.44, 135.43, 149.84 ppm). The following abbreviations are used to designate multiplicities: s = singlet, d = doublet, t = triplet, q = quartet, m = multiplet, br = broad. Low-resolution mass spectra were obtained using a Shimadzu LCMS-2010EV under ESI positive ion mode. High-resolution mass spectra were obtained using a Bruker maXis 4G under ESI positive ion mode.

Compound 3: N-(cyclohex-2-en-1-yl)aniline

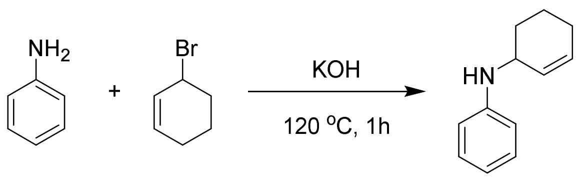

1 2 3

To compound **1** (1 mL, 8.69 mmol) were added compound **2** (0.97 g, 10.4 mmol) and KOH (973 mg, 17.4 mmol). The resulting mixture was stirred at 120 °C for 1 hour, and then quenched with water. The aqueous layer was extracted with EA (3*5 mL). The combined organic layer was washed with saturated NaCl solution, dried over Na_2SO_4, and concentrated under vacuum. The residue was purified by flash chromatography (PE/EA = 3:1) to give compound **3** (1.52 g, 99%).

3： ^{1}H NMR (400 MHz, $CDCl_3$) δ 7.23–7.11 (m, 2H), 6.70 (t, *J* = 7.3 Hz, 1H), 6.66–6.59 (m, 2H), 5.90–5.81 (m, 1H), 5.80–5.72 (m, 1H), 4.00 (s, 1H), 3.92–3.52 (br, 1H), 2.12–1.97 (m, 2H), 1.97–1.83 (m, 1H), 1.73 (m, 1H), 1.63 (m, 2H).

Compound 4: 1',2',3',4'-tetrahydro-[1,1'-biphenyl]-2-amine

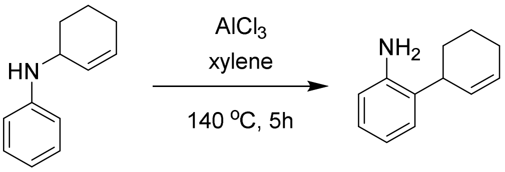

3 4

To a solution of compound **3** (1.00 g, 5.77 mmol) in xylene (5 mL) was added $AlCl_3$ (154 mg, 1.15 mmol). The resulting mixture was stirred at 140 °C for 5 hours, and then quenched with water. The aqueous layer was extracted with EA (3*5 mL). The combined organic layer was washed with saturated NaCl solution, dried over Na_2SO_4, and concentrated under vacuum. The residue was purified by flash chromatography (PE/EA = 10:1) to give compound **4** (430 mg, 43%) as a light yellow solid.

4： ^{1}H NMR (400 MHz, $CDCl_3$) δ 7.15–7.01 (m, 2H), 6.76 (td, *J* = 7.4, 1.1 Hz, 1H), 6.71–6.68 (m, 1H), 5.96 (m, 1H), 5.78–5.70 (m, 1H), 3.76 (br, 2H), 3.43 (m, 1H), 2.18–2.07 (m, 2H), 2.02–1.90 (m, 1H), 1.82–1.57 (m, 3H).

Compound 5: 2-cyclohexylaniline

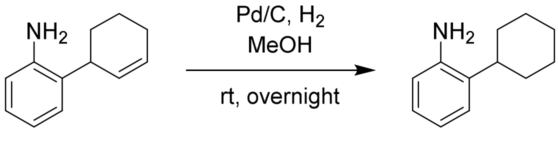

4 **5**

To a solution of compound **4** (100 mg, 0.580 mmol) in MeOH (5 mL) was added palladium 10% on activated carbon (68 mg, 5% equiv.). The mixture was stirred under hydrogen atmosphere overnight. The mixture was filtered by celite and concentrated under vacuum. The residue was purified by flash chromatography (DCM/MeOH = 10:1) to give compound **5** (95 mg, 94%) as a light yellow solid.

5：^{1}H NMR (400 MHz, $CDCl_3$) δ 7.12 (dd, J = 7.7, 1.2 Hz, 1H), 7.02 (td, J = 7.7, 1.4 Hz, 1H), 6.79 (td, J = 7.6, 1.1 Hz, 1H), 6.69 (dd, J = 7.9, 1.1 Hz, 1H), 3.71–3.57 (br, 2H), 2.48 (m, 1H), 1.98–1.82 (m, 4H), 1.78 (m, 1H), 1.50–1.35 (m, 5H).

Compound 7:
4-chloro-N-(1',2',3',4'-tetrahydro-[1,1'-biphenyl]-2-yl)-1,3,5-triazin-2-amine

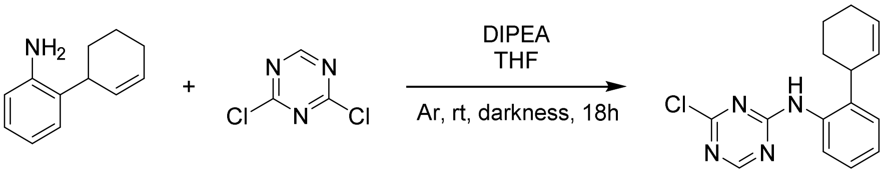

4 **6** **7**

To a solution of compound **6** (173 mg, 1.15 mmol) in THF (5 mL) was added compound **4** (100 mg, 0.577 mmol) under argon atmosphere in darkness. Then DIPEA (1.5 mL) was added dropwise. The resulting mixture was stirred at room temperature for 18 hours under argon atmosphere in darkness. After completion, the reaction was quenched with saturated $NaHCO_3$ solution. The aqueous layer was extracted with DCM (3*5 mL). The combined organic layer was washed with saturated NaCl solution, dried over Na_2SO_4 and concentrated under vacuum. The residue was purified by flash chromatography (PE/EA = 3:1) to give compound **7** (40 mg, 24%) as a light yellow solid.

7：^{1}H NMR (400 MHz, $CDCl_3$) δ 8.37 (s, 1H), 7.82 (m, 1H), 7.67–7.42 (m, 1H), 7.21 (m, 3H), 5.94 (m, 1H), 5.61 (m, 1H), 3.56–3.39 (m, 1H), 2.03 (s, 2H), 1.94–1.81 (m, 1H), 1.67 (s, 1H), 1.60–1.38 (m, 2H).

Compound 8: 4-chloro-N-(2-cyclohexylphenyl)-1,3,5-triazin-2-amine

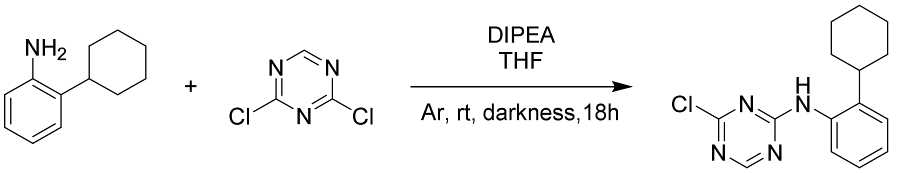

To a solution of compound **6** (162 mg, 1.08 mmol) in THF (5 mL) was added compound **5** (100 mg, 0.577 mmol) under argon atmosphere in darkness. Then DIPEA (1.5 mL) was added dropwise. The resulting mixture was stirred at room temperature for 18 hours under argon atmosphere in darkness. After completion, the reaction was quenched with saturated $NaHCO_3$ solution. The aqueous layer was extracted with DCM (3*5 mL). The combined organic layer was washed with saturated NaCl solution, dried over Na_2SO_4 and concentrated under vacuum. The residue was purified by flash chromatography (PE/EA = 3:1) to give compound **8** (40 mg, 24%) as a light yellow solid.

8: ^{1}H NMR (500 MHz, $CDCl_3$) δ 8.44 (s, 1H), 7.93–7.67 (m, 1H), 7.57–7.39 (m, 1H), 7.38–7.33 (m, 1H), 7.33–7.27 (m, 1H), 7.24 (m, 1H), 2.65 (m, 1H), 1.89–1.70 (m, 5H), 1.51–1.18 (m, 5H).

LRMS (ESI-TOF) m/z: $[M + H]^+$ calculated for $C_{15}H_{18}ClN_4$ 289.12, found 289.20.

Compound 10: 4-chloro-N-(2-(isopropylsulfonyl)phenyl)-1,3,5-triazin-2-amine

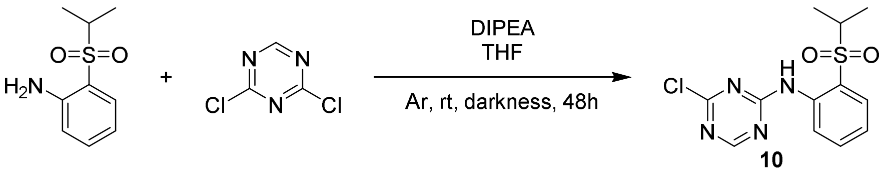

To a solution of 2,4-dichloro-1,3,5-triazine (300 mg, 2.00 mmol) in THF (10 mL) was added 2-(isopropylsulfonyl)aniline (362 mg, 1.82 mmol) under argon atmosphere in darkness. Then DIPEA (1.0 mL) was added dropwise. The resulting mixture was stirred at room temperature for 48 hours under argon. After completion, the reaction was quenched with saturated $NaHCO_3$ solution. The aqueous layer was extracted with DCM (3*10 mL). The combined organic layer was washed with saturated NaCl solution, dried over Na_2SO_4 and concentrated under vacuum. The residue was purified

by flash chromatography (PE/EA = 3:1) to give compound **10** (200 mg, 35%) as a white solid.

10: ^{1}H NMR (400 MHz, $CDCl_3$) δ 9.87 (s, 1H), 8.60 (s, 1H), 8.48 (d, *J* = 8.4 Hz, 1H), 7.91 (dd, *J* = 7.9, 1.1 Hz, 1H), 7.76–7.66 (m, 1H), 7.32 (t, *J* = 7.5 Hz, 1H), 3.21 (m, 1H), 1.30 (d, *J* = 6.8 Hz, 6H).

LRMS (ESI-TOF) m/z: $[M + H]^+$ calculated for $C_{12}H_{14}ClN_4O_2S^+$ 313.05, found 313.05

Compound 12: 4-chloro-N-(2,5-dimethoxyphenyl)-1,3,5-triazin-2-amine

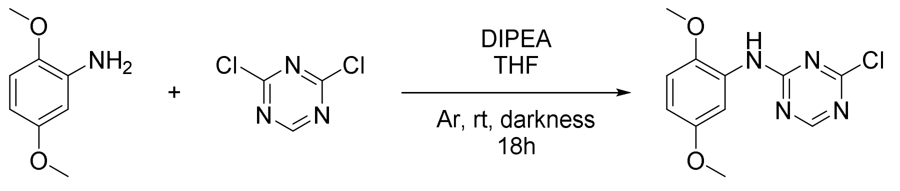

6 **12**

To a solution of 2,5-dimethoxyaniline (185 mg, 1.21 mmol) in THF (5 mL) was added compound **6** (200 mg, 1.33 mmol) under argon atmosphere in darkness. Then DIPEA (0.75 mL) was added dropwise. The resulting mixture was stirred at room temperature for 18 hours under argon atmosphere in darkness. After completion, the reaction was quenched with saturated $NaHCO_3$ solution. The aqueous layer was extracted with DCM (3*5 mL). The combined organic layer was washed with saturated NaCl solution, dried over Na_2SO_4 and concentrated under vacuum. The residue was purified by flash chromatography (PE/EA = 3:1) to give compound **12** (310 mg, 96%) as a white solid.

12: ^{1}H NMR (500 MHz, $CDCl_3$) δ 8.55 (s, 1H), 8.12 (d, *J* = 3.0 Hz, 1H), 6.84 (d, *J* = 8.9 Hz, 1H), 6.63 (dd, *J* = 8.9, 3.0 Hz, 1H), 3.87 (s, 3H), 3.82 (s, 3H).

Compound 31:
N2-(2-methoxy-4-(4-(4-methylpiperazin-1-yl)piperidin-1-yl)phenyl)-N4-(1',2',3',4'-tetrahydro-[1,1'-biphenyl]-2-yl)-1,3,5-triazine-2,4-diamine

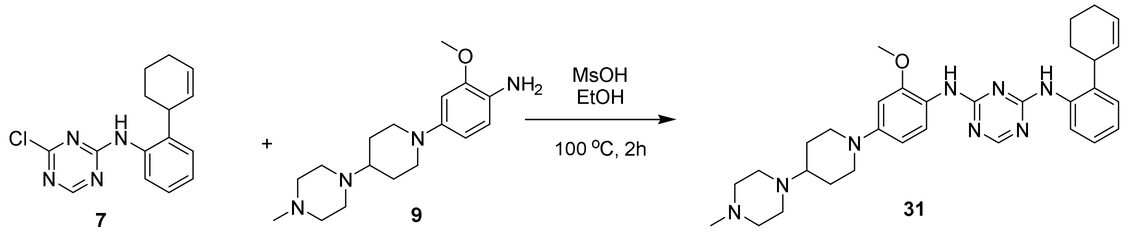

To a solution of compound **7** (40 mg, 0.139 mmol) in EtOH (2 mL) was added compound **9** (55 mg, 0.181 mmol). Then MsOH (36 μL, 0.556 mmol) was added dropwise. The resulting mixture was stirred at 100 °C for 2 hours. After completion, the reaction was quenched with water. The aqueous layer was extracted with EA (3*5 mL). The combined organic layer was washed with saturated NaCl solution, dried over Na_2SO_4 and concentrated under vacuum. The residue was purified by flash chromatography (PE/EA = 5:1) to give compound **31** (2 mg, 3%).

31：^{1}H NMR (500 MHz, $CDCl_3$) δ 8.29 (s, 1H), 8.08 (s, 1H), 7.81 (d, *J* = 8.4 Hz, 1H), 7.47 (s, 1H), 7.29–7.27 (m, 1H), 7.26–7.25 (m, 1H), 7.17 (t, *J* = 7.1 Hz, 1H), 6.51 (d, *J* = 2.0 Hz, 1H), 6.05–6.00 (m, 1H), 5.71 (d, *J* = 9.7 Hz, 1H), 3.85 (s, 3H), 3.66 (d, *J* = 11.8 Hz, 2H), 3.61 (d, *J* = 5.3 Hz, 1H), 2.68 (m, 6H), 2.36 (s, 3H), 2.15–2.07 (m, 2H), 2.02–1.90 (m, 4H), 1.70 (m, 6H), 1.66–1.54 (m, 3H).

HRMS (ESI-TOF) m/z: $[M + Na]^+$ calculated for $C_{32}H_{42}N_8ONa$ 577.3374, found 577.3365.

Compound 32: N2-(2-cyclohexylphenyl)-N4-(2-methoxy-4-(4-(4-methylpiperazin-1-yl)piperidin-1-yl)phenyl)-1,3,5-triazine-2,4-diamine

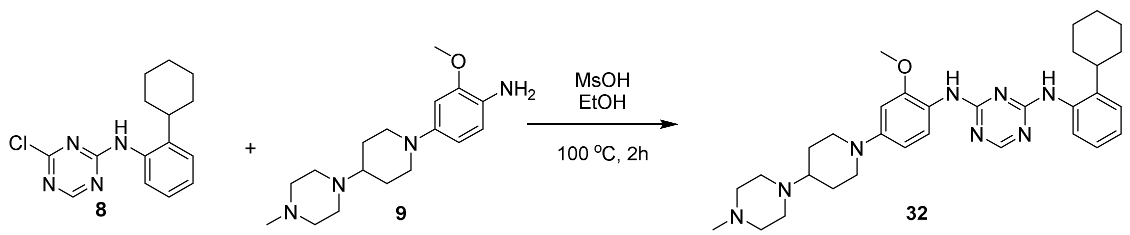

To a solution of compound **8** (100 mg, 0.347 mmol) in EtOH (2 mL) was added compound **9** (137 mg, 0.451 mmol). Then MsOH (75 μL, 1.17 mmol) was added

dropwise. The resulting mixture was stirred at 100 °C for 2 hours. After completion, the reaction was quenched with water. The aqueous layer was extracted with EA (3*5 mL). The combined organic layer was washed with saturated NaCl solution, dried over Na_2SO_4 and concentrated under vacuum. The residue was purified by flash chromatography (PE/EA = 5:1) to give compound **32** (45 mg, 23%) as a white solid.

32: ^{1}H NMR (500 MHz, $CDCl_3$) δ 8.27 (s, 1H), 8.05 (s, 1H), 7.65 (s, 1H), 7.47 (s, 1H), 7.35–7.28 (m, 1H), 7.25–7.20 (m, 2H), 6.50 (s, 1H), 3.83 (s, 3H), 3.64 (d, *J* = 11.4 Hz, 2H), 2.78–2.59 (m, 6H), 2.51 (s, 3H), 2.38 (t, *J* = 11.4 Hz, 1H), 2.31 (s, 3H), 2.17 (s, 2H), 1.94 (d, *J* = 12.1 Hz, 2H), 1.81 (d, *J* = 10.9 Hz, 4H), 1.69 (m, 3H), 1.50–1.31 (m, 4H), 1.31–1.19 (m, 1H).

HRMS (ESI-TOF) m/z: $[M + H]^+$ calculated for $C_{32}H_{45}N_8O^+$ 557.3711, found 557.3712.

Compound 33:
N2-(2-(isopropylsulfonyl)phenyl)-N4-(2-methoxy-5-(trifluoromethyl)phenyl)-1,3,5-triazine-2,4-diamine

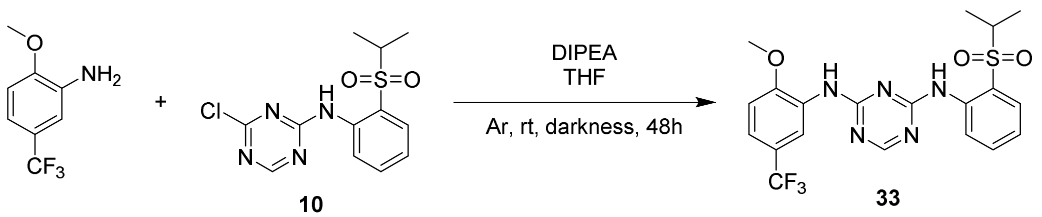

To a solution of compound **10** (50 mg, 0.16 mmol) in THF (3 mL) was added 2-methoxy-5-(trifluoromethyl)aniline (46 mg, 0.24 mmol) under argon atmosphere in darkness. Then DIPEA (0.75 mL) was added dropwise. The resulting mixture was stirred at room temperature for 48 hours under argon. After completion, the reaction was quenched with saturated $NaHCO_3$ solution. The aqueous layer was extracted with DCM (3*5 mL). The combined organic layer was washed with saturated NaCl solution, dried over Na_2SO_4 and concentrated under vacuum. The residue was purified by flash chromatography (PE/EA = 3:1) to give compound **33** (5 mg, 7%).

33: ^{1}H NMR (500 MHz, $CDCl_3$) δ 9.45 (s, 1H), 8.71 (s, 1H), 8.46 (s, 2H), 7.91 (dd, *J* = 7.9, 1.3 Hz, 2H), 7.66 (t, *J* = 7.4 Hz, 1H), 7.33 (dd, *J* = 8.4 Hz, 1.8 Hz, 1H), 7.28 (t,

J = 7.7 Hz, 1H), 6.97 (d, J = 8.5 Hz, 1H), 3.98 (s, 3H), 3.31–3.20 (m, 1H), 1.32 (d, J = 6.8 Hz, 6H).

HRMS (ESI-TOF) m/z: $[M + H]^+$ calculated for $C_{20}H_{21}F_3N_5O_3S^+$ 468.1312, found 468.1316.

Compound 34: methyl 3-((4-((2-(isopropylsulfonyl)phenyl)amino)-1,3,5-triazin-2-yl)amino)-4-methoxybenzoate

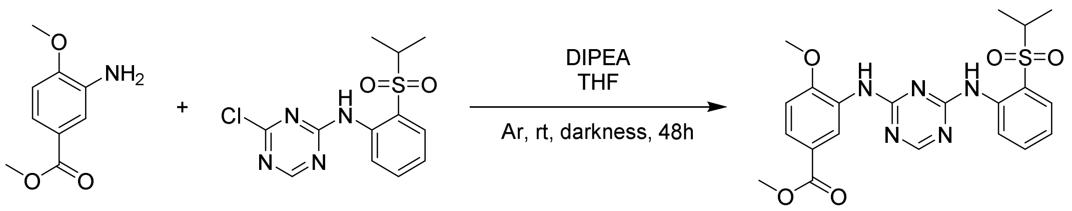

To a solution of compound **10** (50 mg, 0.16 mmol) in THF (3 mL) was added methyl 3-amino-4-methoxybenzoate (43 mg, 0.24 mmol) under argon atmosphere in darkness. Then DIPEA (0.75 mL) was added dropwise. The resulting mixture was stirred at room temperature for 48 hours under argon. After completion, the reaction was quenched with saturated $NaHCO_3$ solution. The aqueous layer was extracted with DCM (3*5 mL). The combined organic layer was washed with saturated NaCl solution, dried over Na_2SO_4 and concentrated under vacuum. The residue was purified by flash chromatography (PE/EA = 2:1) to give compound **34** (14 mg, 19%) as a white solid.

34: ^{1}H NMR (500 MHz, $CDCl_3$) δ 9.51 (s, 1H), 9.03 (s, 1H), 8.59 (d, J = 8.4 Hz, 1H), 8.46 (s, 1H), 7.89 (d, J = 7.8 Hz, 1H), 7.81 (dd, J = 8.5, 1.5 Hz, 1H), 7.73 (s, 1H), 7.63 (t, J = 7.8 Hz, 1H), 7.22 (t, J = 7.6 Hz, 1H), 6.95 (d, J = 8.6 Hz, 1H), 3.97 (s, 3H), 3.88 (s, 3H), 3.31–3.20 (m, 1H), 1.31 (d, J = 6.9 Hz, 6H).

HRMS (ESI-TOF) m/z: $[M + H]^+$ calculated for $C_{21}H_{24}N_5O_5S^+$ 458.1493, found 458.1496.

Compound 35: N2-(2,4-dimethoxyphenyl)-N4-(2-(isopropylsulfonyl)phenyl)-1,3,5-triazine-2,4-diamine

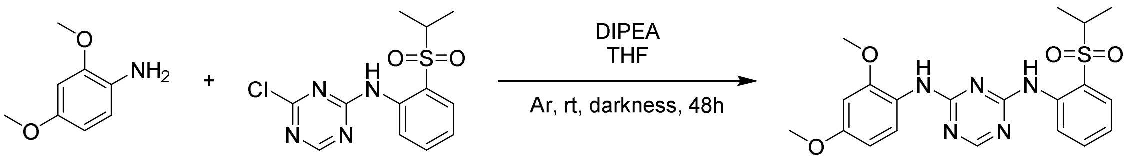

10 35

To a solution of compound **10** (50 mg, 0.16 mmol) in THF (3 mL) was added 2,4-dimethoxyaniline (37 mg, 0.24 mmol) under argon atmosphere in darkness. Then DIPEA (0.75 mL) was added dropwise. The resulting mixture was stirred at room temperature for 48 hours under argon. After completion, the reaction was quenched with saturated $NaHCO_3$ solution. The aqueous layer was extracted with DCM (3*5 mL). The combined organic layer was washed with saturated NaCl solution, dried over Na_2SO_4 and concentrated under vacuum. The residue was purified by flash chromatography (PE/EA = 2:1) to give compound **35** (20 mg, 29%).

35: ^{1}H NMR (500 MHz, $CDCl_3$) δ 9.31 (s, 1H), 8.49 (s, 1H), 8.36 (s, 1H), 8.10 (d, J = 8.8 Hz, 1H), 7.87 (d, J = 7.9 Hz, 1H), 7.61 (s, 2H), 7.22 (t, J = 7.4 Hz, 1H), 6.53–6.47 (m, 2H), 3.87 (s, 3H), 3.81 (s, 3H), 3.24 (m, 1H), 1.30 (d, J = 6.8 Hz, 6H).

HRMS (ESI-TOF) m/z: $[M + H]^+$ calculated for $C_{20}H_{24}N_5O_4S^+$ 430.1544, found 430.1550.

Compound 36: N2-(2,5-dimethoxyphenyl)-N4-(2-(isopropylsulfonyl)phenyl)-1,3,5-triazine-2,4-diamine

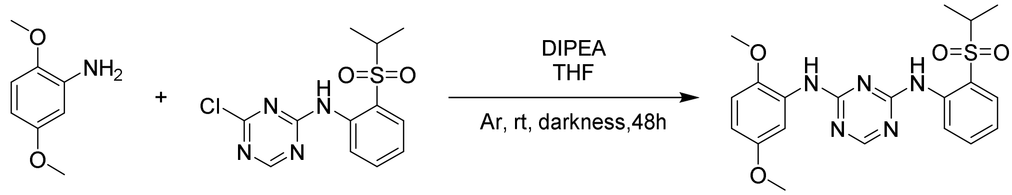

10 36

To a solution of compound **10** (50 mg, 0.16 mmol) in THF (3 mL) was added 2,5-dimethoxyaniline (37 mg, 0.24 mmol) under argon atmosphere in darkness. Then DIPEA (0.75 mL) was added dropwise. The resulting mixture was stirred at room temperature for 48 hours under argon. After completion, the reaction was quenched with saturated $NaHCO_3$ solution. The aqueous layer was extracted with DCM (3*5

mL). The combined organic layer was washed with saturated NaCl solution, dried over Na_2SO_4 and concentrated under vacuum. The residue was purified by flash chromatography (PE/EA = 2:1) to give compound **36** (22 mg, 32%) as a white solid.

36: ^{1}H NMR (400 MHz, $CDCl_3$) δ 9.45 (s, 1H), 8.57 (d, *J* = 8.4 Hz, 1H), 8.44 (s, 1H), 8.17 (s, 1H), 7.88 (dd, *J* = 7.9, 1.2 Hz, 2H), 7.70–7.61 (m, 1H), 7.23 (t, *J* = 7.6 Hz, 1H), 6.82 (d, *J* = 8.9 Hz, 1H), 6.65–6.53 (m, 1H), 3.85 (s, 3H), 3.79 (s, 3H), 3.25 (m, 1H), 1.30 (d, *J* = 6.8 Hz, 6H). ^{13}C NMR (75 MHz, $CDCl_3$) δ 166.7, 163.7, 163.5, 153.8, 142.7, 138.5, 135.0, 131.3, 128.2, 124.2, 123.2, 123.1, 110.9, 107.8,107.1, 60.5, 56.4, 55.8, 15.4.

HRMS (ESI-TOF) m/z: $[M + Na]^+$ calculated for $C_{20}H_{23}N_5O_4SNa^+$ 452.1363, found 452.1369.

Compound 37: N2,N4-bis(2-(isopropylsulfonyl)phenyl)-1,3,5-triazine-2,4-diamine

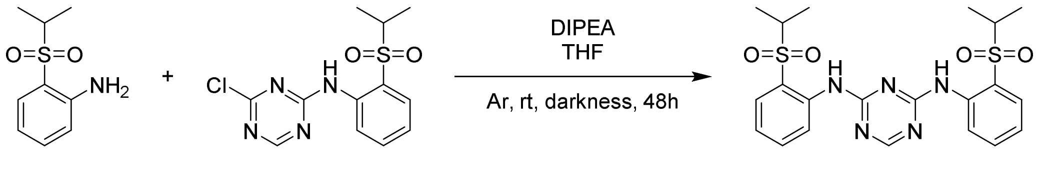

To a solution of compound **10** (50 mg, 0.16 mmol) in THF (3 mL) was added 2-(isopropylsulfonyl)aniline (42 mg, 0.24 mmol) under argon atmosphere in darkness. Then DIPEA (0.75 mL) was added dropwise. The resulting mixture was stirred at room temperature for 48 hours under argon. After completion, the reaction was quenched with saturated $NaHCO_3$ solution. The aqueous layer was extracted with DCM (3*5 mL). The combined organic layer was washed with saturated NaCl solution, dried over Na_2SO_4 and concentrated under vacuum. The residue was purified by flash chromatography (PE/EA = 2:1) to give compound **37** (10 mg, 13%).

37: ^{1}H NMR (400 MHz, $CDCl_3$) δ 9.48 (s, 1H), 8.52 (d, *J* = 8.5 Hz, 2H), 8.02–7.79 (m, 2H), 7.65 (t, *J* = 7.9 Hz, 2H), 7.31–7.23 (m, 2H), 5.03 (s, 2H), 3.31–3.18 (m, 2H), 1.31 (d, *J* = 6.85 Hz, 12H).

HRMS (ESI-TOF) m/z: $[M + H]^+$ calculated for $C_{21}H_{26}N_5O_4S_2^+$ 476.1421, found 476.1426.

Compound 38: N2-(2-(isopropylsulfonyl)phenyl)-N4-(2-methoxyphenyl)-1,3,5-triazine-2,4-diamine

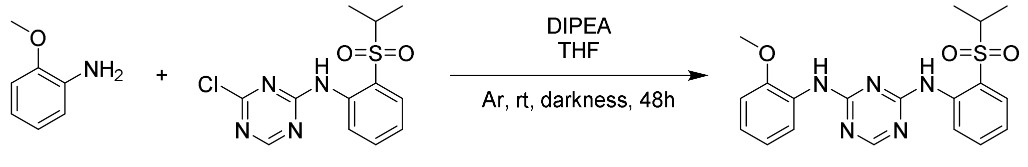

To a solution of compound **10** (30 mg, 0.096 mmol) in THF (3 mL) was added 2-methoxyaniline (18 mg, 0.14 mmol) under argon atmosphere in darkness. Then DIPEA (0.75 mL) was added dropwise. The resulting mixture was stirred at room temperature for 48 hours under argon. After completion, the reaction was quenched with saturated $NaHCO_3$ solution. The aqueous layer was extracted with DCM (3*5 mL). The combined organic layer was washed with saturated NaCl solution, dried over Na_2SO_4 and concentrated under vacuum. The residue was purified by flash chromatography (PE/EA = 3:1) to give compound **38** (22 mg, 48%).

38: ^{1}H NMR (400 MHz, $CDCl_3$) δ 9.34 (s, 1H), 8.52 (d, *J* = 8.1 Hz, 1H), 8.43 (s, 1H), 8.37–8.31 (m, 1H), 7.89 (dd, *J* = 8.0, 1.5 Hz, 2H), 7.64 (t, *J* = 7.8 Hz, 1H), 7.28–7.20 (m, 1H), 7.07 (td, *J* = 7.8, 1.6 Hz, 1H), 6.98 (t, *J* = 7.7 Hz, 1H), 6.94–6.90 (m, 1H), 3.91 (s, 3H), 3.33–3.18 (m, 1H), 1.31 (d, *J* = 6.9 Hz, 6H).

HRMS (ESI-TOF) m/z: $[M + H]^+$ calculated for $C_{19}H_{22}N_5O_3S^+$ 400.1438, found 400.1442.

Compound 39: N2-(2-(isopropylsulfonyl)phenyl)-N4-(2-methoxy-5-methylphenyl)-1,3,5-triazine-2,4-diamine

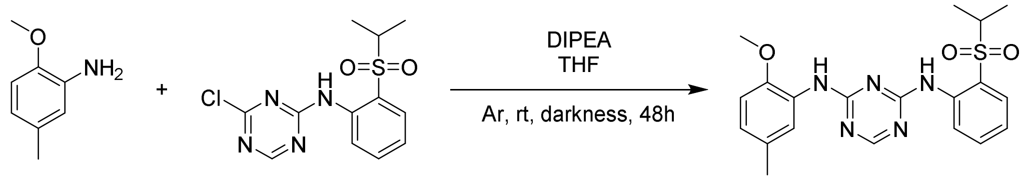

To a solution of compound **10** (30 mg, 0.096 mmol) in THF (3 mL) was added 2-methoxy-5-methylaniline (20 mg, 0.14 mmol) under argon atmosphere in darkness. Then DIPEA (0.75 mL) was added dropwise. The resulting mixture was stirred at

room temperature for 48 hours under argon. After completion, the reaction was quenched with saturated $NaHCO_3$ solution. The aqueous layer was extracted with DCM (3*5 mL). The combined organic layer was washed with saturated NaCl solution, dried over Na_2SO_4 and concentrated under vacuum. The residue was purified by flash chromatography (PE/EA = 2:1) to give a solid compound **39** (22 mg, 57%).

39: ^{1}H NMR (400 MHz, $CDCl_3$) δ 9.35 (s, 1H), 8.54 (d, *J* = 8.3 Hz, 1H), 8.43 (s, 1H), 8.16 (s, 1H), 7.89 (dd, *J* = 7.9, 1.1 Hz, 1H), 7.82 (s, 1H), 7.64 (t, *J* = 7.3 Hz, 1H), 7.27–7.20 (m, 1H), 6.91–6.71 (m, 2H), 3.87 (s, 3H), 3.34–3.17 (m, 1H), 2.30 (s, 3H), 1.31 (d, *J* = 6.9 Hz, 6H).

HRMS (ESI-TOF) m/z: $[M + H]^+$ calculated for $C_{20}H_{24}N_5O_3S^+$ 414.1594, found 414.1598.

Compound 40: N2-(2-(isopropylsulfonyl)phenyl)-N4-(2-methoxy-5-nitrophenyl) -1,3,5-triazine-2,4-diamine

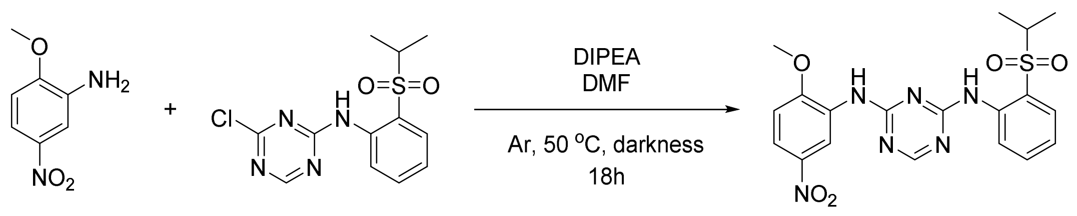

10 **40**

To a solution of compound **10** (30 mg, 0.096 mmol) in DMF (3 mL) was added 2-methoxy-5-nitroaniline (32 mg, 0.19 mmol) under argon atmosphere in darkness. Then DIPEA (0.50 mL) was added dropwise. The resulting mixture was stirred at 50 °C for 18 hours under argon. After completion, the reaction was quenched with saturated $NaHCO_3$ solution. The aqueous layer was extracted with DCM (3*5 mL). The combined organic layer was washed with saturated NaCl solution, dried over Na_2SO_4 and concentrated under vacuum. The residue was purified by flash chromatography (PE/EA = 3:1) to give compound **40** (9 mg, 19%).

40: ^{1}H NMR (500 MHz, $CDCl_3$) δ 9.48 (s, 1H), 8.75–8.64 (m, 1H), 8.61–8.53 (m, 1H), 8.49–8.44 (m, 1H), 8.05–7.99 (m, 1H), 7.95–7.90 (m, 1H), 7.82–7.77 (m, 1H), 7.68 (m, 1H), 4.06–4.03 (m, 3H), 3.28–3.22 (m, 1H), 1.32 (m, 6H).

HRMS (ESI-TOF) m/z: $[M + H]^+$ calculated for $C_{19}H_{21}N_6O_5S^+$ 445.1289, found 445.1293.

Compound 41: N2-(5-fluoro-2-methoxyphenyl)-N4-(2-(isopropylsulfonyl)phenyl)-1,3,5-triazine-2,4-diamine

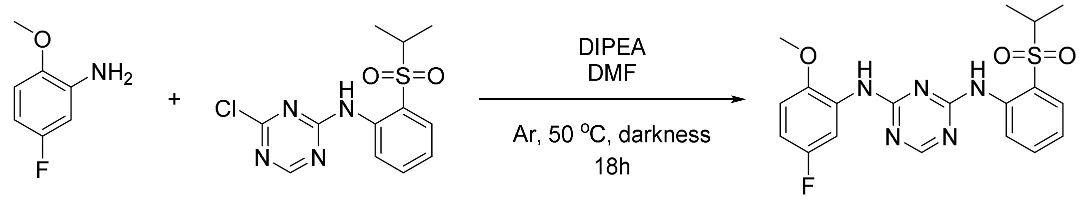

10 **41**

To a solution of compound **10** (30 mg, 0.096 mmol) in DMF (2.5 mL) was added 5-fluoro-2-methoxyaniline (20 mg, 0.14 mmol) under argon atmosphere in darkness. Then DIPEA (0.75 mL) was added dropwise. The resulting mixture was stirred at 50 °C for 18 hours under argon. After completion, the reaction was quenched with saturated $NaHCO_3$ solution. The aqueous layer was extracted with DCM (3*5 mL). The combined organic layer was washed with saturated NaCl solution, dried over Na_2SO_4 and concentrated under vacuum. The residue was purified by flash chromatography (PE/EA = 3:1) to give compound **41** (2 mg, 5%).

41: ^{1}H NMR (400 MHz, $CDCl_3$) δ 9.49 (s, 1H), 8.55–8.49 (m, 1H), 8.38 (s, 1H), 8.14 (dd, *J* = 10.6, 3.0 Hz, 1H), 7.95–7.86 (m, 1H), 7.67 (m, 2H), 7.31 (t, *J* =7.7 Hz, 1H), 6.83 (dd, *J* = 9.0, 4.9 Hz, 1H), 6.79–6.71 (m, 1H), 3.92 (s, 3H), 3.30–3.17 (m, 1H), 1.31 (d, *J* = 7.1 Hz, 6H).

LRMS (ESI-TOF) m/z: $[M + H]^+$ calculated for $C_{19}H_{21}FN_5O_3S^+$ 418.14, found 418.20.

Compound 42: N2-(2-(isopropylsulfonyl)phenyl)-N4-(2-methoxy-5-(trifluoromethoxy)phenyl)-1,3,5-triazine-2,4-diamine

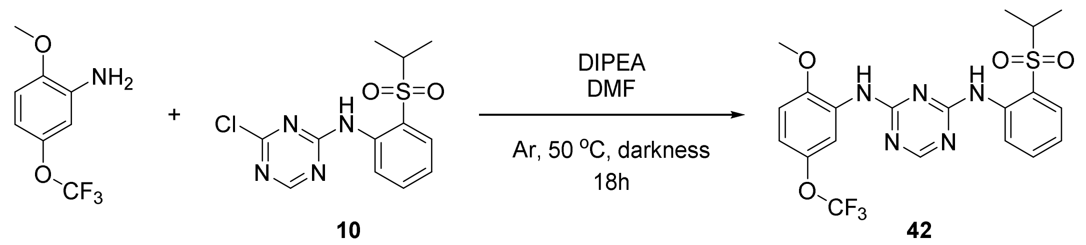

To a solution of compound **10** (40 mg, 0.13 mmol) in DMF (2.5 mL) was added 2-methoxy-5-(trifluoromethoxy)aniline (40 mg, 0.19 mmol) under argon atmosphere in darkness. Then DIPEA (0.50 mL) was added dropwise. The resulting mixture was stirred at 50 °C for 18 hours under argon. After completion, the reaction was quenched with saturated $NaHCO_3$ solution. The aqueous layer was extracted with DCM (3*5 mL). The combined organic layer was washed with saturated NaCl solution, dried over Na_2SO_4 and concentrated under vacuum. The residue was purified by flash chromatography (PE/EA = 3:1) to give compound **42** (10 mg, 16%) as a gray solid.

42： ^{1}H NMR (400 MHz, $CDCl_3$) δ 9.33 (s, 1H), 7.53–7.36 (m, 2H), 7.91 (d, *J* = 7.7 Hz, 1H), 7.87–7.77 (m, 1H), 7.68 (t, *J* = 7.5 Hz, 1H), 7.35–7.23 (m, 2H), 6.95–6.83 (m, 2H), 3.93 (s, 3H), 3.28–3.20 (m, 1H), 1.30 (d, *J* = 6.8 Hz, 6H).

HRMS (ESI-TOF) m/z: $[M + H]^+$ calculated for $C_{20}H_{21}F_3N_5O_4S^+$ 484.1261, found 484.1264.

Compound 38a: N2-(2-(isopropylsulfonyl)phenyl)-N4-(2-methoxypyridin-3-yl) -1,3,5-triazine-2,4-diamine

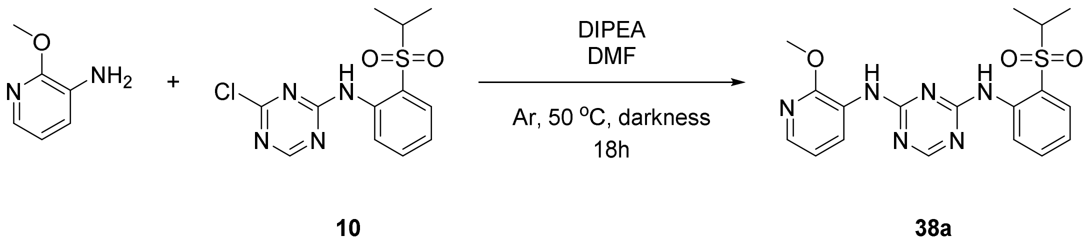

To a solution of compound **10** (30 mg, 0.096 mmol) in DMF (3 mL) was added 2-methoxypyridin-3-amine (18 mg, 0.14 mmol) under argon atmosphere in darkness. Then DIPEA (0.75 mL) was added dropwise. The resulting mixture was stirred at 50 °C for 18 hours under argon. After completion, the reaction was quenched with

saturated $NaHCO_3$ solution. The aqueous layer was extracted with DCM (3*5 mL). The combined organic layer was washed with saturated NaCl solution, dried over Na_2SO_4 and concentrated under vacuum. The residue was purified by flash chromatography (PE/EA = 3:1) to give compound **38a** (2 mg, 5%).

38a: ^{1}H NMR (500 MHz, $CDCl_3$) δ 9.38 (s, 1H), 8.67–8.52 (m, 2H), 8.48 (m, 2H), 7.92–7.87 (d, *J* = 7.8 Hz, 1H), 7.86 (m, 1H), 7.65 (m, 1H), 7.62–7.57 (m, 1H), 6.96–6.86 (m, 1H), 4.09–4.00 (m, 3H), 3.30–3.23 (m, 1H), 1.32 (d, *J* = 6.6 Hz, 1H). HRMS (ESI-TOF) m/z: $[M + H]^+$ calculated for $C_{18}H_{21}N_6O_3S^+$ 401.1390, found 401.1397.

Compound 38b: N2-(2-(isopropylsulfonyl)phenyl)-N4-(3-methoxypyridin-2-yl) -1,3,5-triazine-2,4-diamine

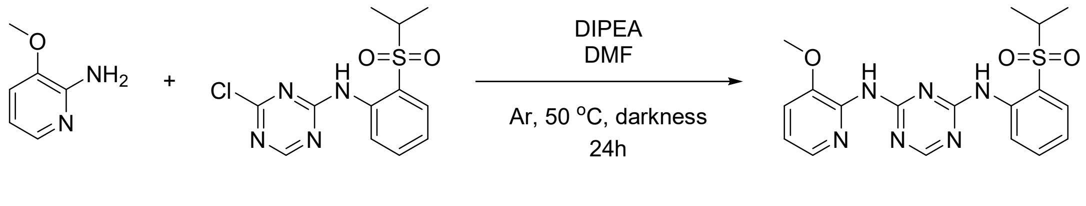

10 **38b**

To a solution of compound **10** (30 mg, 0.096 mmol) in DMF (2.5 mL) was added 3-methoxypyridin-2-amine (18 mg, 0.14 mmol) under argon atmosphere in darkness. Then DIPEA (0.75 mL) was added dropwise. The resulting mixture was stirred at 50 °C for 24 hours under argon. After completion, the reaction was quenched with saturated $NaHCO_3$ solution. The aqueous layer was extracted with DCM (3*5 mL). The combined organic layer was washed with saturated NaCl solution, dried over Na_2SO_4 and concentrated under vacuum. The residue was purified by flash chromatography (PE/EA = 3:1) to give compound **38b** (7 mg, 18%).

38b: ^{1}H NMR (500 MHz, $CDCl_3$) δ 9.28 (s, 1H), 8.52 (d, *J* = 8.4 Hz, 1H), 8.34 (s, 1H), 7.86 (d, *J* = 7.9 Hz, 1H), 7.68–7.63 (m, 1H), 7.21 (t, *J* = 7.7 Hz, 1H), 6.90 (d, *J* = 7.8 Hz, 1H), 6.61 (dd, *J* = 7.8, 5.1 Hz, 1H), 5.60 (s, 1H), 4.74 (s, 1H), 3.83 (s, 3H), 3.31–3.18 (m, 1H), 1.30 (d, *J* = 6.8 Hz, 6H).

HRMS (ESI-TOF) m/z: $[M + H]^+$ calculated for $C_{18}H_{21}N_6O_3S^+$ 401.1390, found 401.1397.

Compound 36a: N2-(2,5-dimethoxypyridin-3-yl)-N4-(2-(isopropylsulfonyl)phenyl)-1,3,5-triazine-2,4-diamine

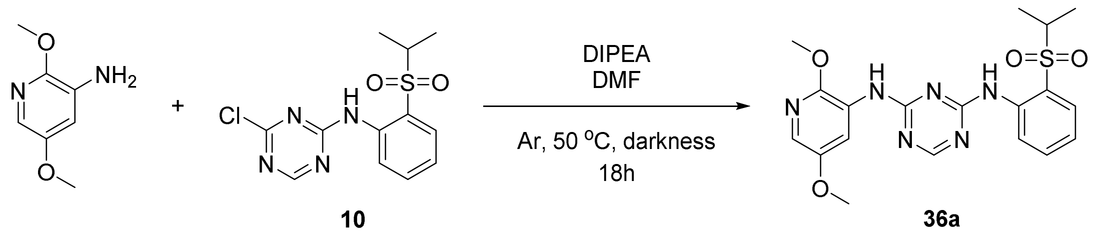

To a solution of compound **10** (90 mg, 0.29 mmol) in DMF (3 mL) was added 2,5-dimethoxypyridin-3-amine (49 mg, 0.32 mmol) under argon atmosphere in darkness. Then DIPEA (0.5 mL) was added dropwise. The resulting mixture was stirred at 50 °C for 18 hours under argon. After completion, the reaction was quenched with saturated $NaHCO_3$ solution. The aqueous layer was extracted with DCM (3*5 mL). The combined organic layer was washed with saturated NaCl solution, dried over Na_2SO_4 and concentrated under vacuum. The residue was purified by flash chromatography (PE/EA = 3:1) to give compound **36a** (34 mg, 27%) as a white solid.

36a：^{1}H NMR (500 MHz, $CDCl_3$) δ 9.47 (s, 1H), 8.52 (d, J = 8.2 Hz, 1H), 8.47 (m, 1H), 8.00 (s, 1H), 7.91–7.83 (m, 1H), 7.64 (m, 2H), 7.49 (d, J = 2.6 Hz, 1H), 7.27–7.21 (m, 1H), 3.98 (s, 3H), 3.82 (s, J = 7.1 Hz, 3H), 3.28–3.18 (m, 1H), 1.29 (d, J = 6.9 Hz, 6H).

HRMS (ESI-TOF) m/z: $[M + H]^+$ calculated for $C_{19}H_{23}N_6O_4S^+$ 431.1496, found 431.1502.

Compound 36b': 6-chloro-N-(2-(isopropylsulfonyl)phenyl)pyridin-2-amine

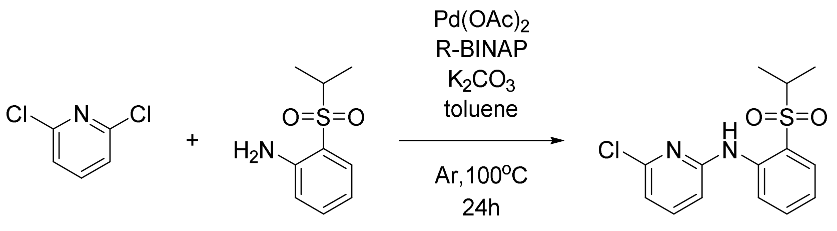

36b'

To a solution of palladium acetate (14 mg, 0.060 mmol) in toluene (10 mL) was added R-BINAP (38 mg, 0.060 mmol) under argon atmosphere and anhydrous conditions. After stirring at room temperature for 10 minutes, the palladium reagent needed for the reaction was prepared. Then, 2-(isopropylsulfonyl)aniline (239 mg, 1.20 mmol) and K_2CO_3 (2.76 g, 20.0 mmol) were added to toluene (5 mL) solution of 2,6-dichloropyridine (148 mg, 1.00 mmol) under the protection of argon in anhydrous condition. After mixing well, the prepared palladium reagent solution was added dropwise. The resulting mixture was heated to 100 °C under argon and stirred for 24 hours. After the reaction is complete, the solution was filtered with celite, washed with DCM, and concentrated under vacuum. The residue was purified by flash chromatography (PE/EA = 3:1) to give compound **36b'** (34 mg, 27%) as a gray-white solid.

36b': ^{1}H NMR (500 MHz, $CDCl_3$) δ 8.99 (s, 1H), 8.39 (d, J = 7.8 Hz, 1H), 7.77 (d, J = 7.0 Hz, 1H), 7.55 (m, 1H), 7.48 (m, 1H), 7.07 (m, 1H), 6.82 (d, J = 6.4 Hz, 1H), 6.68 (d, J = 7.4 Hz, 1H), 3.35–3.07 (m, 1H), 1.25 (d, J = 5.1 Hz, 6H).

Compound 36b:

N2-(2,5-dimethoxyphenyl)-N6-(2-(isopropylsulfonyl)phenyl)pyridine -2,6-diamine

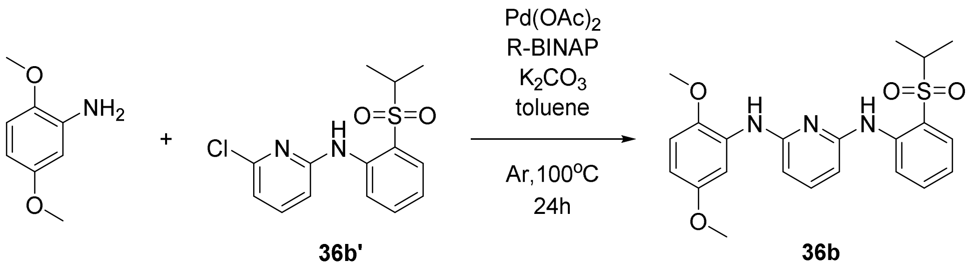

36b' **36b**

To a solution of palladium acetate (6 mg, 0.027 mmol) in toluene (6 mL) was added R-BINAP (17 mg, 0.027 mmol) under argon atmosphere and anhydrous conditions.

After stirring at room temperature for 10 minutes, the palladium reagent needed for the reaction was prepared. Then, **36b'** (120 mg, 0.453 mmol) and K_2CO_3 (1.25 g, 9.06 mmol) were added to toluene (3 mL) solution of 2,5-dimethoxyaniline (77 mg, 0.50 mmol) under the protection of argon in anhydrous condition. After mixing well, the prepared palladium reagent solution was added dropwise. The resulting mixture was heated to 100 °C under argon and stirred for 24 hours. After the reaction is complete, the solution was filtered with celite, washed with DCM, and concentrated under vacuum. The residue was purified by flash chromatography (PE/EA = 3:1) to give compound **36b** (42 mg, 22%) as a light yellow solid.

36b: ^{1}H NMR (500 MHz, $CDCl_3$) δ 8.68 (s, 1H), 8.29 (d, *J* = 8.4 Hz, 1H), 7.84 (d, *J* = 3.0 Hz, 1H), 7.82 (dd, *J* = 8.0, 1.4 Hz, 1H), 7.56–7.50 (m, 1H), 7.43 (t, *J* = 7.9 Hz, 1H), 7.04 (t, *J* = 7.6 Hz, 1H), 6.99 (s, 1H), 6.79 (d, *J* = 8.8 Hz, 1H), 6.45 (dd, *J* = 8.8, 3.0 Hz, 1H), 6.42 (d, *J* = 8.0 Hz, 1H), 6.33 (d, *J* = 7.8 Hz, 1H), 3.86 (s, 3H), 3.63 (s, 3H), 3.35–3.27 (m, 1H), 1.30 (d, *J* = 6.9 Hz, 6H); ^{13}C NMR (126 MHz, $CDCl_3$) δ 154.2, 153.9, 152.8, 142.5, 141.4, 139.2, 134.9, 131.2, 131.0, 122.0, 120.6, 120.4, 110.7, 105.2, 105.1, 102.6, 101.9, 56.2, 55.6, 54.8, 15.3.

HRMS (ESI-TOF) m/z: $[M + H]^+$ calculated for $C_{22}H_{26}N_3O_4S^+$ 428.1639, found 428.1647.

Compound 36c': 6-chloro-N-(2,5-dimethoxyphenyl)pyrazin-2-amine

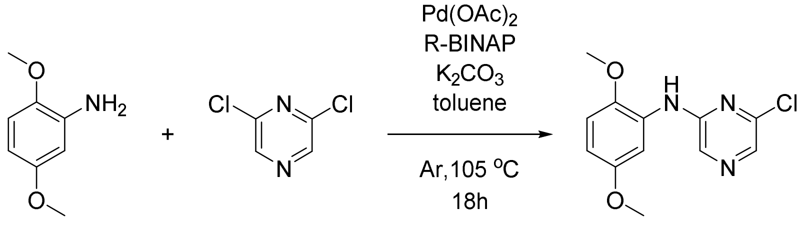

36c'

To a solution of palladium acetate (9 mg, 0.04 mmol) in toluene (7.5 mL) was added R-BINAP (24 mg, 0.04 mmol) under argon atmosphere and anhydrous conditions. After stirring at room temperature for 10 minutes, the palladium reagent needed for the reaction was prepared. Then, 2,5-dimethoxyaniline (100 mg, 0.65 mmol) and K_2CO_3 (898 mg, 6.5 mmol) were added to toluene (7.5 mL) solution of

2,6-dichloropyrazine (116 mg, 0.78 mmol) under the protection of argon in anhydrous condition. After mixing well, the prepared palladium reagent solution was added dropwise. The resulting mixture was heated to 105 °C under argon and stirred for 18 hours. After the reaction is complete, the solution was filtered with celite, washed with DCM, and concentrated under vacuum. The residue was purified by flash chromatography (PE/EA = 4:1) to give compound **36c'** (120 mg, 69%).

36c'：^{1}H NMR (300 MHz, $CDCl_3$) δ 8.08 (s, 1H), 7.95 (s, 1H), 7.94 (s, 1H), 7.29 (s, 1H), 6.81 (d, *J* = 8.9 Hz, 1H), 6.54 (dd, *J* = 8.9, 3.0 Hz, 1H), 3.85 (s, 3H), 3.80 (s, 3H).

Compound 36c:

N2-(2,5-dimethoxyphenyl)-N6-(2-(isopropylsulfonyl)phenyl)pyrazine-2,6-diamine

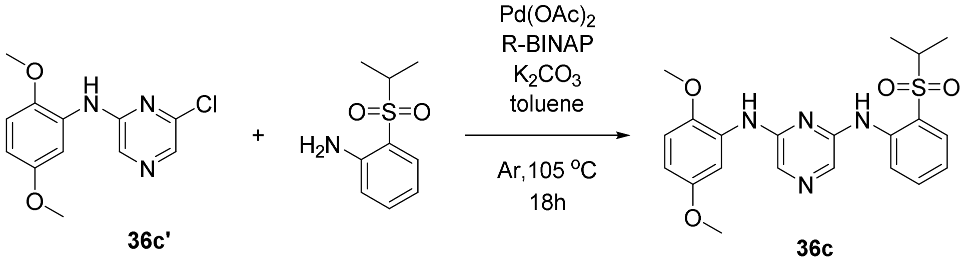

To a solution of palladium acetate (6 mg, 0.026 mmol) in toluene (5 mL) was added R-BINAP (16 mg, 0.026 mmol) under argon atmosphere and anhydrous conditions. After stirring at room temperature for 10 minutes, the palladium reagent needed for the reaction was prepared. Then, 2-(isopropylsulfonyl)aniline (54 mg, 0.27 mmol) and K_2CO_3 (312 mg, 2.26 mmol) were added to toluene (5 mL) solution of **36c'** (60 mg, 0.226 mmol) under the protection of argon in anhydrous condition. After mixing well, the prepared palladium reagent solution was added dropwise. The resulting mixture was heated to 105 °C under argon and stirred for 18 hours. After the reaction is complete, the solution was filtered with celite, washed with DCM, and concentrated under vacuum. The residue was purified by flash chromatography (PE/EA = 2:1) to give compound **36c** (45 mg, 46%).

36c：^{1}H NMR (300 MHz, $CDCl_3$) δ 8.89 (s, 1H), 8.31 (d, *J* = 8.4 Hz, 1H), 7.90–7.82 (m, 2H), 7.79 (s, 1H), 7.70 (s, 1H), 7.64–7.51 (m, 1H), 7.16–7.08 (m, 2H), 6.81 (d, *J*

= 8.8 Hz, 1H), 6.49 (dd, J = 8.8, 2.9 Hz, 1H), 3.87 (s, 3H), 3.65 (s, 3H), 3.34–3.17 (m, 1H), 1.30 (d, J = 6.9 Hz, 6H); ^{13}C NMR (101 MHz, $CDCl_3$) δ 154.0, 149.9, 148.6, 142.8, 140.5, 135.2, 131.5, 129.9, 125.2, 123.3, 122.9, 121.6, 121.0, 110.8, 106.0, 105.9, 56.3, 55.7, 55.5, 15.4.

HRMS (ESI-TOF) m/z: $[M + H]^+$ calculated for $C_{21}H_{25}N_4O_4S^+$ 429.1591, found 429.1596.

Compound 36d': N-(3-chlorophenyl)-2,5-dimethoxyaniline

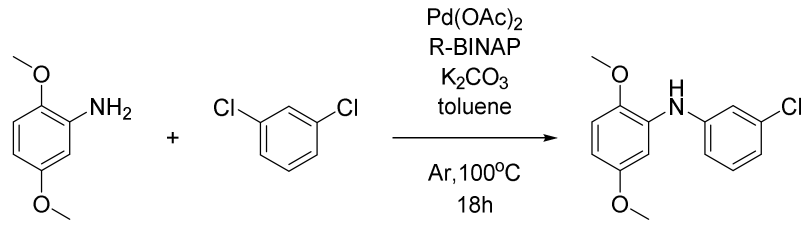

36d'

To a solution of palladium acetate (9 mg, 0.04 mmol) in toluene (7.5 mL) was added R-BINAP (24 mg, 0.040 mmol) under argon atmosphere and anhydrous conditions. After stirring at room temperature for 10 minutes, the palladium reagent needed for the reaction was prepared. Then, 2,5-dimethoxyaniline (100 mg, 0.650 mmol) and K_2CO_3 (898 mg, 6.50 mmol) were added to toluene (7.5 mL) solution of 1,3-dichlorobenzene (115 mg, 0.780 mmol) under the protection of argon in anhydrous condition. After mixing well, the prepared palladium reagent solution was added dropwise. The resulting mixture was heated to 100 °C under argon and stirred for 18 hours. After the reaction is complete, the solution was filtered with celite, washed with DCM, and concentrated under vacuum. The residue was purified by flash chromatography (PE/EA = 5:1) to give compound **36d'** (107 mg, 62%).

36d': ^{1}H NMR (300 MHz, $CDCl_3$) δ 7.21 (s, 1H), 7.19–7.15 (m, 1H), 7.09–7.01 (m, 1H), 6.96–6.88 (m, 2H), 6.83 (d, J = 8.8 Hz, 1H), 6.43 (dd, J = 8.8, 2.9 Hz, 1H), 6.28–6.17 (br, 1H), 3.87 (s, 3H), 3.78 (s, 3H).

Compound 36d:
N1-(2,5-dimethoxyphenyl)-N3-(2-(isopropylsulfonyl)phenyl)benzene-1,3-diamine

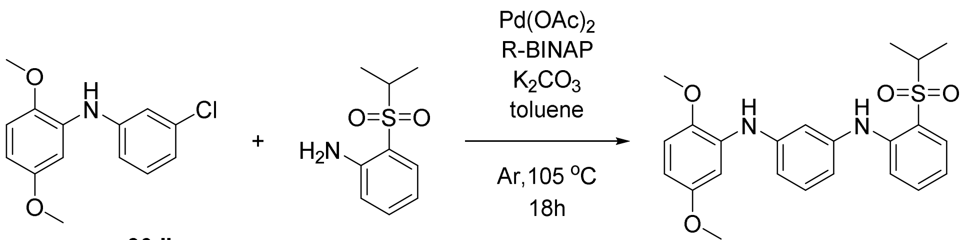

36d' **36d**

To a solution of palladium acetate (9 mg, 0.04 mmol) in toluene (5 mL) was added R-BINAP (24 mg, 0.040 mmol) under argon atmosphere and anhydrous conditions. After stirring at room temperature for 10 minutes, the palladium reagent needed for the reaction was prepared. Then, 2-(isopropylsulfonyl)aniline (64 mg, 0.32 mmol) and K_2CO_3 (440 mg, 3.18 mmol) were added to toluene (5 mL) solution of **36d’** (70 mg, 0.265 mmol) under the protection of argon in anhydrous condition. After mixing well, the prepared palladium reagent solution was added dropwise. The resulting mixture was heated to 105 °C under argon and stirred for 18 hours. After the reaction is complete, the solution was filtered with celite, washed with DCM, and concentrated under vacuum. The residue was purified by flash chromatography (PE/EA = 2:1) to give compound **36d** (10 mg, 9%).

36d：^{1}H NMR (300 MHz, $CDCl_3$) δ 7.91 (s, 1H), 7.81–7.74 (m, 1H), 7.43–7.39 (m, 2H), 7.23 (t, J = 7.8 Hz, 1H), 6.94 (t, J = 2.2 Hz, 1H), 6.92 (d, J = 2.9 Hz, 2H), 6.90–6.87 (m, 1H), 6.79 (d, J = 8.8 Hz, 1H), 6.75–6.71 (m, 1H), 6.37 (dd, J = 8.7, 2.9 Hz, 1H), 6.19 (s, 1H), 3.84 (s, 3H), 3.74 (s, 3H), 3.42–3.31 (m, 1H), 1.32 (d, J = 6.9 Hz, 6H).^{13}C NMR (101 MHz, $CDCl_3$) δ 154.2, 144.5, 143.8, 142.9, 141.7, 135.1, 133.5, 131.9, 130.5, 121.1, 119.2, 116.8, 114.0, 113.7, 111.3, 110.9, 103.5, 102.5, 56.3, 55.8, 54.3, 15.5.

Compound 36e’: 6-chloro-N-(2,5-dimethoxyphenyl)pyrimidin-4-amine

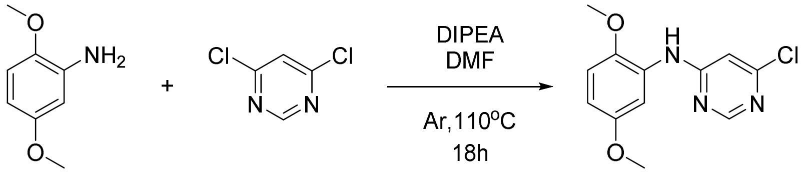

36e'

To a solution of 4,6-dichloropyrimidine (232 mg, 1.56 mmol) in DMF (3 mL) was added 2,5-dimethoxyaniline (200 mg, 1.30 mmol) under argon atmosphere. Then DIPEA (0.75 mL) was added dropwise. The resulting mixture was stirred at 110 °C for 18 hours under argon. After completion, the reaction was quenched with saturated $NaHCO_3$ solution. The aqueous layer was extracted with DCM (3*5 mL). The combined organic layer was washed with saturated NaCl solution, dried over Na_2SO_4 and concentrated under vacuum. The residue was purified by flash chromatography (PE/EA = 3:1) to give compound **36e'** (335 mg, 97%).

36e': ^{1}H NMR (400 MHz, $CDCl_3$) δ 8.48 (s, 1H), 7.61 (s, 1H), 7.47 (s, 1H), 6.83 (d, *J* = 8.9 Hz, 1H), 6.70 (s, 1H), 6.61 (dd, *J* = 8.9, 3.0 Hz, 1H), 3.81 (s, 3H), 3.78 (s, 3H).

Compound 36e:
N4-(2,5-dimethoxyphenyl)-N6-(2-(isopropylsulfonyl)phenyl)pyrimidine-4,6-diamine

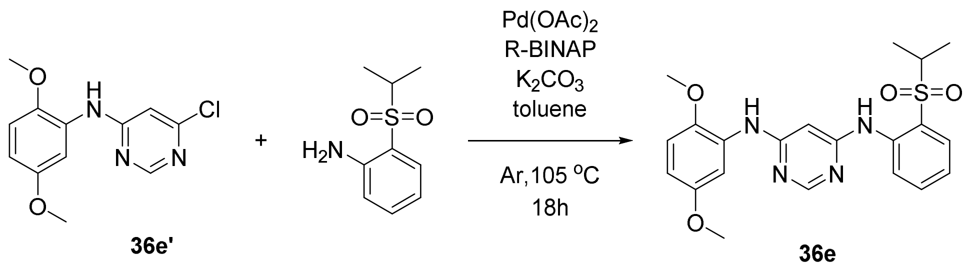

To a solution of palladium acetate (8 mg, 0.036 mmol) in toluene (5 mL) was added R-BINAP (22 mg, 0.036 mmol) under argon atmosphere and anhydrous conditions. After stirring at room temperature for 10 minutes, the palladium reagent needed for the reaction was prepared. Then, 2-(isopropylsulfonyl)aniline (45 mg, 0.226 mmol) and K_2CO_3 (414 mg, 3.01 mmol) were added to toluene (5 mL) solution of **36e'** (40 mg, 0.150 mmol) under the protection of argon in anhydrous condition. After mixing well, the prepared palladium reagent solution was added dropwise. The resulting mixture was heated to 105 °C under argon and stirred for 18 hours. After the reaction is complete, the solution was filtered with celite, washed with DCM, and concentrated under vacuum. The residue was purified by flash chromatography (PE/EA = 2:1) to give compound **36e** (23 mg, 36%).

36e: ^{1}H NMR (400 MHz, $CDCl_3$) δ 8.77 (s, 1H), 8.46 (s, 1H), 8.30–8.24 (m, 1H), 7.87 (dd, *J* = 8.0, 1.6 Hz, 1H), 7.68–7.58 (m, 2H), 7.23–7.15 (m, 2H), 6.87 (d, *J* = 8.9 Hz, 1H), 6.61 (dd, *J* = 8.9, 3.0 Hz, 1H), 6.18 (s, 1H), 3.87 (s, 3H), 3.83 (s, 3H), 3.31–3.20 (m, 1H), 1.30 (d, *J* = 6.8 Hz, 6H).

HRMS (ESI-TOF) m/z: $[M + H]^+$ calculated for $C_{21}H_{25}N_4O_4S^+$ 429.1591, found 429.1592.

Compound 36f': 4-chloro-N-(2,5-dimethoxyphenyl)pyrimidin-2-amine

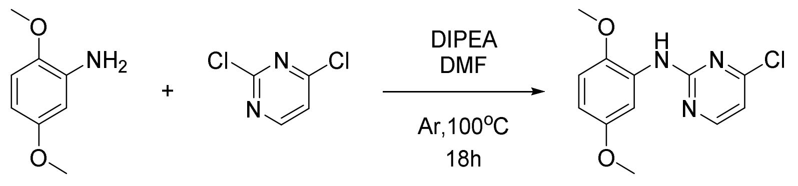

36f'

To a solution of 2,4-dichloropyrimidine (232 mg, 1.56 mmol) in DMF (5 mL) was added 2,5-dimethoxyaniline (200 mg, 1.30 mmol) under argon atmosphere. Then DIPEA (0.75 mL) was added dropwise. The resulting mixture was stirred at 100 °C for 18 hours under argon. After completion, the reaction was quenched with saturated $NaHCO_3$ solution. The aqueous layer was extracted with DCM (3*5 mL). The combined organic layer was washed with saturated NaCl solution, dried over Na_2SO_4 and concentrated under vacuum. The residue was purified by flash chromatography (PE/EA = 3:1) to give compound **36f'** (180 mg, 52%).

36f'： ^{1}H NMR (300 MHz, $CDCl_3$) δ 8.13 (d, *J* = 5.9 Hz, 1H), 7.66 (s, 1H), 7.44 (s, 1H), 6.84 (d, *J* = 8.9 Hz, 1H), 6.66–6.58 (m, 2H), 3.83 (s, 3H), 3.80 (s, 3H).

Compound 36f:
N2-(2,5-dimethoxyphenyl)-N4-(2-(isopropylsulfonyl)phenyl)pyrimidine-2,4-diamine

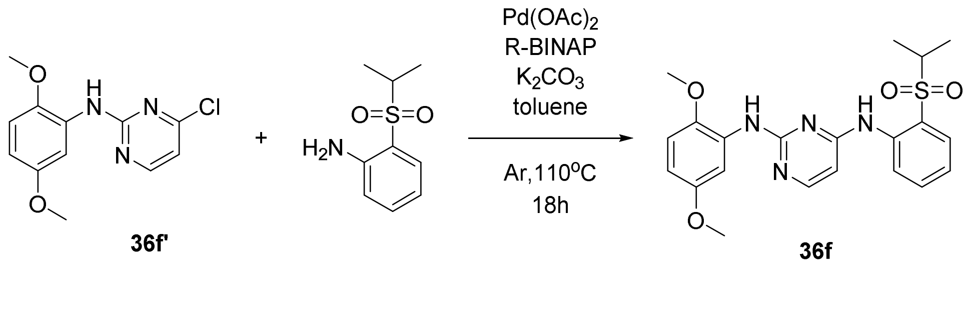

To a solution of palladium acetate (20 mg, 0.088 mmol) in toluene (5 mL) was added R-BINAP (54 mg, 0.088 mmol) under argon atmosphere and anhydrous conditions. After stirring at room temperature for 10 minutes, the palladium reagent needed for the reaction was prepared. Then, 2-(isopropylsulfonyl)aniline (49 mg, 0.24 mmol) and K_2CO_3 (561 mg, 4.06 mmol) were added to toluene (5 mL) solution of **36f'** (54 mg, 0.203 mmol) under the protection of argon in anhydrous condition. After mixing well, the prepared palladium reagent solution was added dropwise. The resulting mixture was heated to 110 °C under argon and stirred for 18 hours. After the reaction is complete, the solution was filtered with celite, washed with DCM, and concentrated under vacuum. The residue was purified by flash chromatography (PE/EA = 1:1) to give compound **36f** (41 mg, 47%).

36f: ^{1}H NMR (400 MHz, $CDCl_3$) δ 9.28 (s, 1H), 8.69 (d, J = 8.5 Hz, 1H), 8.10 (d, J = 5.8 Hz, 1H), 8.03–7.98 (m, 1H), 7.83 (dd, J = 7.9, 1.6 Hz, 1H), 7.66–7.54 (m, 1H), 7.20 (s, 1H), 7.13–7.03 (m, 1H), 6.82 (d, J = 8.9 Hz, 1H), 6.56 (dd, J = 8.8, 3.0 Hz, 1H), 6.28 (d, J = 5.8 Hz, 1H), 3.86 (s, 3H), 3.80 (s, 3H), 3.37–3.23 (m, 1H), 1.30 (d, J = 6.8 Hz, 6H). ^{13}C NMR (101 MHz, $CDCl_3$) δ 160.6, 159.1, 156.5, 153.9, 143.0, 140.4, 135.0, 131.2, 129.0, 122.5, 121.4, 121.3, 111.1, 108.0, 106.6, 100.4, 56.4, 55.9, 55.2, 15.4.

HRMS (ESI-TOF) m/z: $[M + H]^+$ calculated for $C_{21}H_{25}N_4O_4S^+$ 429.1591, found 429.1591.

Compound 36K2b:

dibenzyl(6-((2-((2-((4-((2,5-dimethoxyphenyl)amino)-1,3,5-triazin-2-yl)amino) phenyl)sulfonyl)ethyl)amino)-6-oxohexane-1,5-diyl)(S)-dicarbamate

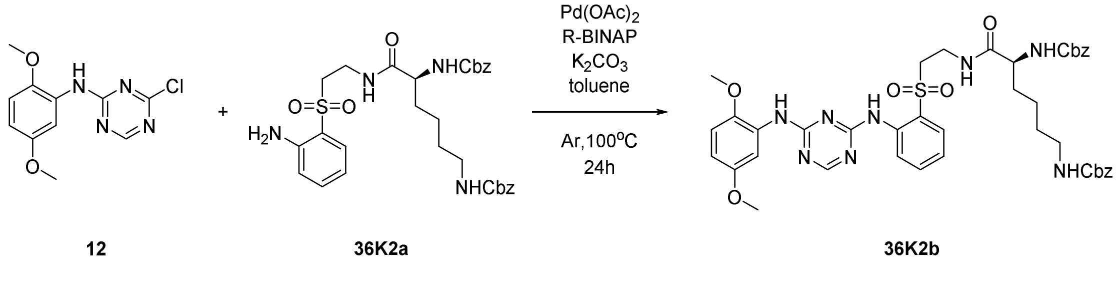

12 **36K2a** **36K2b**

To a solution of palladium acetate (8 mg, 0.036 mmol) in toluene (5 mL) was added

R-BINAP (22 mg, 0.036 mmol) under argon atmosphere and anhydrous conditions. After stirring at room temperature for 10 minutes, the palladium reagent needed for the reaction was prepared. Then, compound **12** (22 mg, 0.082 mmol) and K_2CO_3 (348 mg, 2.52 mmol) were added to toluene (5 mL) solution of **36K2a** (50 mg, 0.084 mmol) under the protection of argon in anhydrous condition. After mixing well, the prepared palladium reagent solution was added dropwise. The resulting mixture was heated to 100 °C under argon and stirred for 24 hours. After the reaction is complete, the solution was filtered with celite, washed with DCM, and concentrated under vacuum. The residue was purified by flash chromatography (PE/EA = 1:3) to give compound **36K2b** (21 mg, 30%).

36K2b: ^{1}H NMR (500 MHz, $CDCl_3$) δ 9.08 (s, 1H), 8.51 (d, *J* = 8.3 Hz, 1H), 8.41 (s, 1H), 8.08 (s, 1H), 7.90 (d, *J* = 7.7 Hz, 1H), 7.35–7.27 (m, 11H), 6.94 (m, 1H), 6.82–6.75 (m, 1H), 6.57 (dd, *J* = 8.9, 3.0 Hz, 1H), 5.83 (s, 1H), 5.06–4.96 (m, 4H), 4.07 (m, 1H), 3.84 (s, 3H), 3.76 (s, 3H), 3.66 (m, 2H), 3.30 (m, 2H), 3.13 (m, 2H), 1.78 (s, 1H), 1.59–1.40 (m, 3H), 1.39–1.29 (m, 3H).

HRMS (ESI-TOF) m/z: $[M + Na]^+$ calculated for $C_{41}H_{46}N_8O_9SNa^+$ 849.3001, found 849.2999.

Compound 36K2:

(S)-2,6-diamino-N-(2-((2-((4-((2,5-dimethoxyphenyl)amino)-1,3,5-triazin-2-yl)amino)phenyl)sulfonyl)ethyl)hexanamide

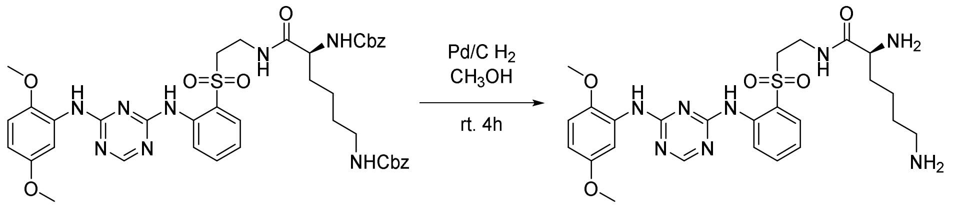

36K2b **36K2**

To a solution of compound **36K2b** (11 mg, 0.013 mmol) in MeOH (2 mL) was added palladium 10% on activated carbon (11 mg). The mixture was stirred under hydrogen atmosphere for 4 hours. The mixture was filtered by celite, washed with MeOH, and concentrated under vacuum. The residue was purified by flash chromatography

(DCM/MeOH = 10:1) to give compound **36K2** (2 mg, 28%).

36K2: ^{1}H NMR (500 MHz, CD_3OD) δ 8.47 (s, 1H), 8.37 (s, 1H), 7.93 (dd, J = 8.0, 1.5 Hz, 1H), 7.77 (s, 1H), 7.70 (m, 1H), 7.59–7.54 (m, 1H), 7.35–7.26 (m, 3H), 6.96 (d, J = 8.9 Hz, 1H), 6.67 (dd, J = 8.9, 3.0 Hz, 1H), 3.86 (s, 3H), 3.75–3.73 (s, 3H), 3.26–3.19 (m, 5H), 2.94 (m, 2H), 1.88 (m, 2H), 1.68 (m, 2H), 1.44 (m, 2H).

LRMS (ESI-TOF) m/z: $[M + H]^+$ calculated for $C_{25}H_{35}N_8O_5S^+$ 559.25, found 559.50.

Compound 36K3b:

dibenzyl(6-((3-((2-((4-((2,5-dimethoxyphenyl)amino)-1,3,5-triazin-2-yl)amino) phenyl)sulfonyl)propyl)amino)-6-oxohexane-1,5-diyl)(S)-dicarbamate

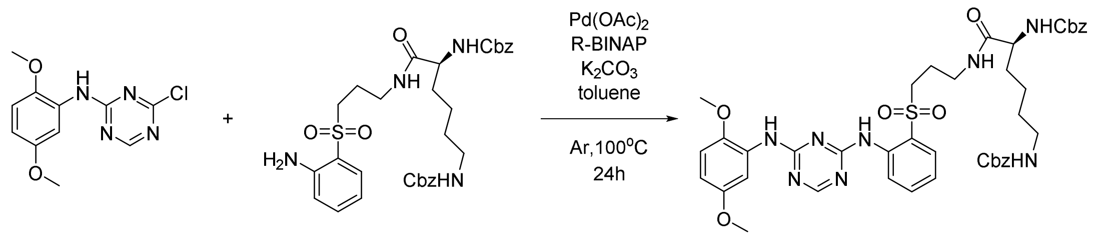

To a solution of palladium acetate (5 mg, 0.021 mmol) in toluene (3 mL) was added R-BINAP (13 mg, 0.021 mmol) under argon atmosphere and anhydrous conditions. After stirring at room temperature for 10 minutes, the palladium reagent needed for the reaction was prepared. Then, compound **12** (21 mg, 0.078 mmol) and K_2CO_3 (180 mg, 1.30 mmol) were added to toluene (3 mL) solution of **36K3a** (60 mg, 0.101 mmol) under the protection of argon in anhydrous condition. After mixing well, the prepared palladium reagent solution was added dropwise. The resulting mixture was heated to 100 °C under argon and stirred for 24 hours. After the reaction is complete, the solution was filtered with celite, washed with DCM, and concentrated under vacuum. The residue was purified by flash chromatography (PE/EA = 1:3) to give compound **36K3b** (27 mg, 39%).

36K3b: ^{1}H NMR (500 MHz, $CDCl_3$) δ 9.22 (s, 1H), 8.55 (d, J = 8.4 Hz, 1H), 8.42 (s, 1H), 8.11 (s, 1H), 7.42 (m, 1H), 7.29 (m, 10H), 6.80 (d, J = 8.9 Hz, 2H), 6.58–6.53 (m, 1H), 5.87–5.60 (m, 2H), 5.05 (m, 4H), 4.05–3.97 (m, 1H), 3.83 (s, 3H), 3.76 (s, 3H),

3.23 (m, 2H), 3.10 (m, 4H), 1.94–1.86 (m, 2H), 1.73 (m, 2H), 1.53 (m, 2H), 1.41 (m, 2H).

LRMS (ESI-TOF) m/z: $[M + H]^+$ calculated for $C_{42}H_{49}N_8O_9S^+$ 841.34, found 841.40.

Compound 36K3:

(S)-2,6-diamino-N-(3-((2-((4-((2,5-dimethoxyphenyl)amino)-1,3,5-triazin-2-yl) amino)phenyl)sulfonyl)propyl)hexanamide

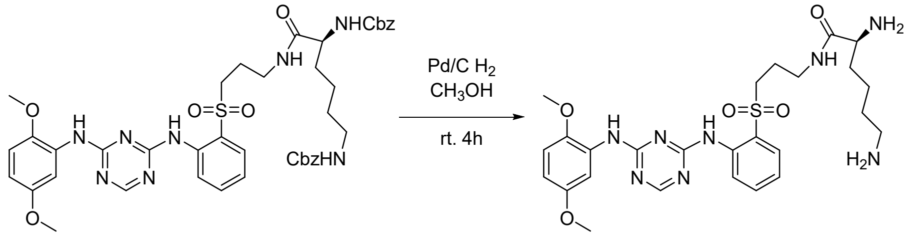

36K3b **36K3**

To a solution of compound **36K3b** (5 mg, 0.008 mmol) in MeOH (3 mL) was added palladium 10% on activated carbon (6 mg). The mixture was stirred under hydrogen atmosphere for 4 hours. The mixture was filtered by celite, washed with MeOH, and concentrated under vacuum. The residue was purified by flash chromatography (DCM/MeOH = 10:1) to give compound **36K3** (2 mg, 73%).

36K3: ^{1}H NMR (600 MHz, CD_3OD) δ 8.50 (s, 1H), 8.37 (s, 1H), 7.93 (d, *J* = 8.0, 1H), 7.77 (s, 1H), 7.71 (s, 1H), 7.35–7.27 (m, 3H), 6.95 (d, *J* = 8.9 Hz, 1H), 6.67 (dd, *J* = 8.9, 3.0 Hz, 1H), 3.86 (s, 3H), 3.73 (s, 3H), 3.25–3.18 (m, 4H) 3.12 (m, 1H), 2.93 (m, 2H), 1.89–1.86 (m, 2H), 1.71–1.65 (m, 2H), 1.48-1.36 (m, 4H). ^{13}C NMR (151 MHz, CD_3OD) δ 167.4, 136.12, 136.06, 131.1, 130.63, 130.62, 129.2, 128.8, 128.73, 128.67, 128.5, 124.9, 118.4, 117.5, 112.2, 109.3, 56.60, 55.95, 54.5, 54.0, 52.2, 39.3, 38.61, 38.55, 23.7, 22.9.

HRMS (ESI-TOF) m/z: $[M + H]^+$ calculated for $C_{26}H_{37}N_8O_5S^+$ 573.2602, found 573.2599.

Compound 36K4b:

dibenzyl(6-((4-((2-((4-((2,5-dimethoxyphenyl)amino)-1,3,5-triazin-2-yl)amino) phenyl)sulfonyl)butyl)amino)-6-oxohexane-1,5-diyl)(S)-dicarbamate

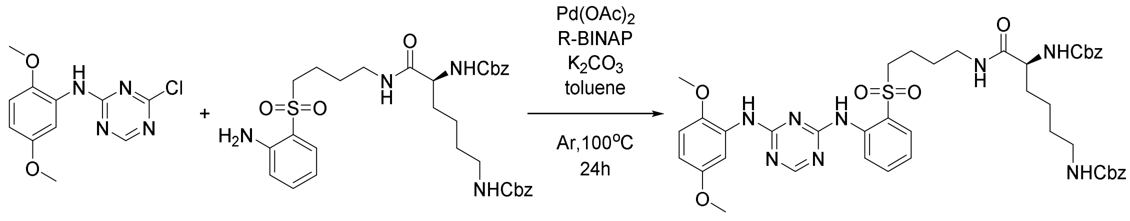

To a solution of palladium acetate (6 mg, 0.027 mmol) in toluene (3 mL) was added R-BINAP (17 mg, 0.027 mmol) under argon atmosphere and anhydrous conditions. After stirring at room temperature for 10 minutes, the palladium reagent needed for the reaction was prepared. Then, compound **12** (22 mg, 0.082 mmol) and K_2CO_3 (101 mg, 0.730 mmol) were added to toluene (3 mL) solution of **36K4a** (43 mg, 0.068 mmol) under the protection of argon in anhydrous condition. After mixing well, the prepared palladium reagent solution was added dropwise. The resulting mixture was heated to 100 °C under argon and stirred for 24 hours. After the reaction is complete, the solution was filtered with celite, washed with DCM, and concentrated under vacuum. The residue was purified by flash chromatography (PE/EA = 1:3) to give compound **36K4b** (11 mg, 19%).

36K4b: ^{1}H NMR (500 MHz, $CDCl_3$) δ 9.24 (s, 1H), 8.53 (d, J = 8.4 Hz, 1H), 8.43 (s, 1H), 8.13 (s, 1H), 7.88 (d, J = 7.9 Hz, 1H), 7.61 (m, 1H), 7.30 (m, 10H), 6.81 (d, J = 8.9 Hz, 1H), 6.77 (m, 1H), 6.57 (dd, J = 8.8, 3.0 Hz, 1H), 6.43 (s, 1H), 5.73 (s, 1H), 5.06–4.99 (m, 4H), 4.01 (m, 1H), 3.84 (s, 3H), 3.77 (s, 3H), 3.12 (m, 6H), 1.93 (m, 2H), 1.72 (m, 4H), 1.59–1.40 (m, 2H), 1.39–1.26 (m, 2H).

HRMS (ESI-TOF) m/z: $[M + Na]^+$ calculated for $C_{43}H_{50}N_8O_9SNa^+$ 877.3314, found 877.3296.

Compound 36K4:

(S)-2,6-diamino-N-(4-((2-((4-((2,5-dimethoxyphenyl)amino)-1,3,5-triazin-2-yl) amino)phenyl)sulfonyl)butyl)hexanamide

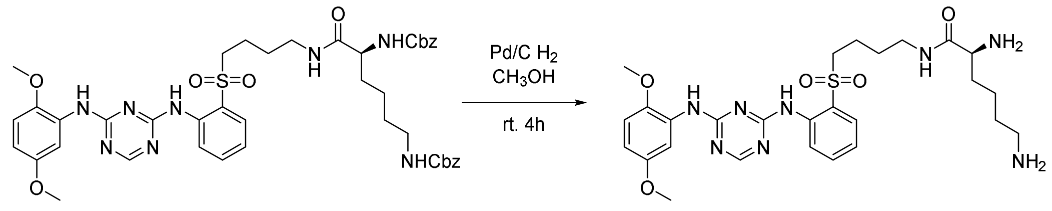

36K4b **36K4**

To a solution of compound **36K4b** (10 mg, 0.011 mmol) in MeOH (5 mL) was added palladium 10% on activated carbon (10 mg). The mixture was stirred under hydrogen atmosphere for 4 hours. The mixture was filtered by celite, washed with MeOH, and concentrated under vacuum. The residue was purified by flash chromatography (DCM/MeOH = 10:1) to give compound **36K4** (4 mg, 62%).

36K4： ^{1}H NMR (500 MHz, CD_3OD) δ 8.41 (s, 1H), 8.36 (s, 1H), 7.97–7.88 (dd, J = 8.0, 1.4 Hz, 1H), 7.76 (s, 1H), 7.68 (m, 1H), 7.50–7.43 (m, 1H), 7.38–7.29 (m, 3H), 6.93 (d, J = 8.9 Hz, 1H), 6.65 (dd, J = 8.9, 3.0 Hz, 1H), 3.85 (s, 3H), 3.71 (s, 3H), 3.29–3.25 (m, 2H), 3.24–3.17 (m, 2H), 3.10 (m, 1H), 2.98–2.91 (m, 2H), 1.80–1.63 (m, 6H), 1.48–1.38 (m, 2H), 1.34–1.23 (m, 2H). ^{13}C NMR (126 MHz, CD_3OD) δ 172.1, 166.1, 163.6, 162.2, 153.6, 150.1, 134.8, 129.9, 127.4, 126.5, 123.9, 120.5, 118.5, 111.0, 108.9, 108.1, 55.4, 54.8, 54.6, 53.6, 39.0, 37.9, 32.4, 27.4, 26.9, 21.9, 19.3.

HRMS (ESI-TOF) m/z: $[M + Na]^+$ calculated for $C_{27}H_{38}N_8O_5SNa^+$ 609.2578, found 609.2587.

Compound 36K4*′ *b:

dibenzyl(6-((3-((2-((4-((2,5-dimethoxyphenyl)amino)-1,3,5-triazin-2-yl)amino) phenyl)sulfonyl)cyclobutyl)amino)-6-oxohexane-1,5-diyl)(S)-dicarbamate

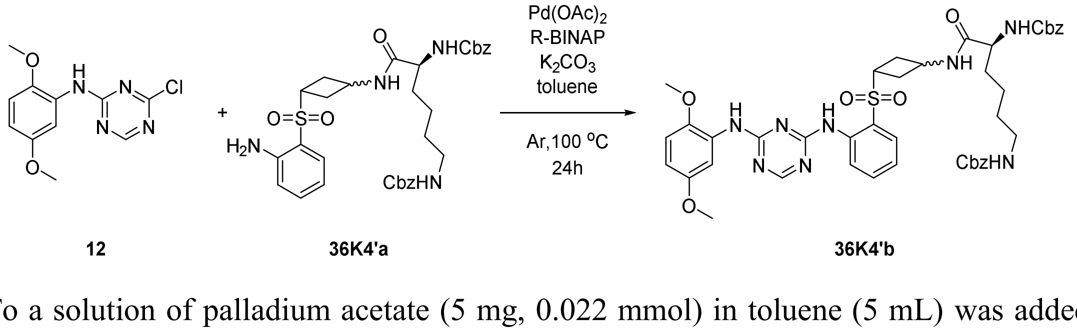

12 **36K4'a** **36K4'b**

To a solution of palladium acetate (5 mg, 0.022 mmol) in toluene (5 mL) was added R-BINAP (14 mg, 0.022 mmol) under argon atmosphere and anhydrous conditions.

After stirring at room temperature for 10 minutes, the palladium reagent needed for the reaction was prepared. Then, compound **12** (21 mg, 0.080 mmol) and K_2CO_3 (221 mg, 1.60 mmol) were added to toluene (3 mL) solution of **36K4'a** (50 mg, 0.080 mmol) under the protection of argon in anhydrous condition. After mixing well, the prepared palladium reagent solution was added dropwise. The resulting mixture was heated to 100 °C under argon and stirred for 24 hours. After the reaction is complete, the solution was filtered with celite, washed with DCM, and concentrated under vacuum. The residue was purified by flash chromatography (PE/EA = 1:3) to give compound **36K4'b** (12 mg, 18%).

36K4'b: ^{1}H NMR (500 MHz, $CDCl_3$) δ 9.29 (s, 1H), 8.57 (m, 1H), 8.42 (s, 1H), 8.13 (s, 1H), 7.90–7.87 (m, 1H), 7.84 (m, 1H), 7.64 (m, 1H), 7.35–7.28 (m, 10H), 7.19 (m, 1H), 6.81 (d, J = 8.9 Hz, 1H), 6.74 (m, 1H), 6.68 (d, J = 8.2 Hz, 1H), 6.57 (m, 1H), 5.67–5.56 (m, 1H), 5.00 (m, 4H), 4.50–4.40 (m, 1H), 4.02 (m, 1H), 3.84 (s, 3H), 3.77 (s, 3H), 3.12 (m, 3H), 2.81 (s, 2H), 2.33–2.16 (m, 2H), 1.77 (m, 2H), 1.46 (m, 2H), 1.32 (m, 2H).

HRMS (ESI-TOF) m/z: $[M + H]^+$ calculated for $C_{43}H_{49}N_8O_9S^+$ 853.3338, found 853.3336.

Compound 36K4':

(S)-2,6-diamino-N-(3-((2-((4-((2,5-dimethoxyphenyl)amino)-1,3,5-triazin-2-yl) amino)phenyl)sulfonyl)cyclobutyl)hexanamide

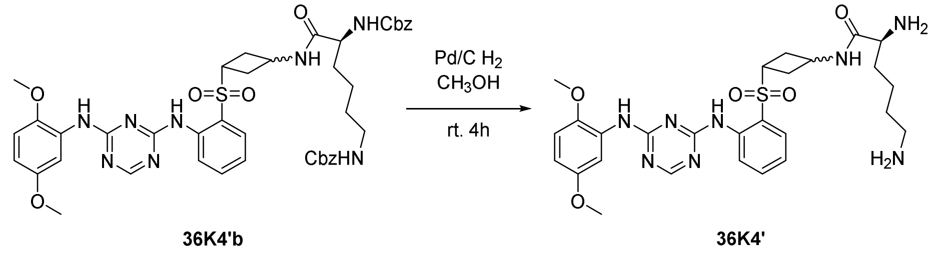

To a solution of compound **36K4'b** (3 mg, 0.004 mmol) in MeOH (2 mL) was added palladium 10% on activated carbon (5 mg). The mixture was stirred under hydrogen atmosphere for 4 hours. The mixture was filtered by celite, washed with MeOH, and concentrated under vacuum. The residue was purified by flash chromatography

(DCM/MeOH = 10:1) to give compound **36K4'** (1 mg, 46%).

36K4': ^{1}H NMR (600 MHz, CD_3OD) δ 8.46–8.36 (m, 1H), 8.33 (s, 1H), 7.92 (d, J = 8.1 Hz, 1H), 7.73 (m, 1H), 7.66 (m, 1H), 7.33-7.26 (m, 3H), 6.92 (m, 1H), 6.63 (m, 1H), 4.18 (m, 1H), 3.82 (s, 3H), 3.68 (s, 3H), 3.51 (m, 1H), 3.21–3.19 (m, 1H), 2.82 (m, 2H), 2.31 (m, 2H), 1.64 (m, 1H), 1.60–1.54 (m, 3H), 1.43–1.38 (m, 2H) 1.32–1.23 (m, 2H). ^{13}C NMR (151 MHz, CD_3OD) δ 178.5, 173.1, 166.2, 160.8, 160.2, 142.9, 134.9, 131.0, 130.3, 128.5, 128.1, 128.0, 125.1, 123.7, 111.1, 108.3, 69.8, 67.7, 65.1, 62.7, 55.5, 38.8, 33.5, 30.2, 29.4, 23.6, 22.6.

HRMS (ESI-TOF) m/z: [M] calculated for $C_{27}H_{36}N_8O_5S$ 584.2529, found 584.2546.

Compound 36K5b:

dibenzyl(6-((5-((2-((4-((2,5-dimethoxyphenyl)amino)-1,3,5-triazin-2-yl)amino) phenyl)sulfonyl)pentyl)amino)-6-oxohexane-1,5-diyl)(S)-dicarbamate

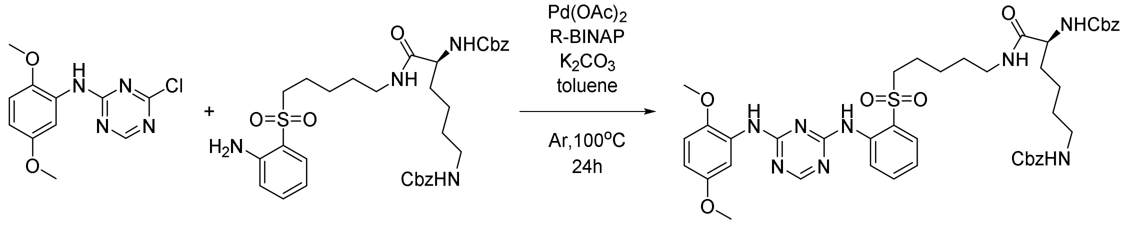

12 **36K5a** **36K5b**

To a solution of palladium acetate (10 mg, 0.044 mmol) in toluene (3 mL) was added R-BINAP (27 mg, 0.044 mmol) under argon atmosphere and anhydrous conditions. After stirring at room temperature for 10 minutes, the palladium reagent needed for the reaction was prepared. Then, compound **12** (32 mg, 0.120 mmol) and K_2CO_3 (414 mg, 3.00 mmol) were added to toluene (3 mL) solution of **36K5a** (64 mg, 0.10 mmol) under the protection of argon in anhydrous condition. After mixing well, the prepared palladium reagent solution was added dropwise. The resulting mixture was heated to 100 °C under argon and stirred for 24 hours. After the reaction is complete, the solution was filtered with celite, washed with DCM, and concentrated under vacuum. The residue was purified by flash chromatography (PE/EA = 1:3) to give compound **36K5b** (12 mg, 14%).

36K5b: ^{1}H NMR (500 MHz, $CDCl_3$) δ 9.22 (s, 1H), 8.49 (d, *J* = 8.3 Hz, 1H), 8.44 (s, 1H), 8.09 (s, 1H), 7.90 (d, *J* = 7.8 Hz, 1H), 7.34–7.26 (m, 10H), 6.57 (dd, *J* = 8.9, 2.9 Hz, 1H), 6.45–6.39 (m, 1H), 6.30 (s, 1H), 6.12–6.05 (m, 1H), 5.85 (m, 1H), 5.65 (d, *J* = 7.4 Hz, 1H), 5.05 (m, 4H), 4.96 (s, 1H), 4.04 (s, 1H), 3.85 (s, 3H), 3.77 (s, 3H), 3.20–3.04 (m, 7H), 1.79 (m, 1H), 1.70 (m, 2H), 1.48 (m, 3H), 1.33 (m, 6H).
HRMS (ESI-TOF) m/z: $[M + Na]^+$ calculated for $C_{44}H_{52}N_8O_9SNa^+$ 891.3470, found 891.3469.

Compound 36K5:

(S)-2,6-diamino-N-(5-((2-((4-((2,5-dimethoxyphenyl)amino)-1,3,5-triazin-2-yl) amino)phenyl)sulfonyl)pentyl)hexanamide

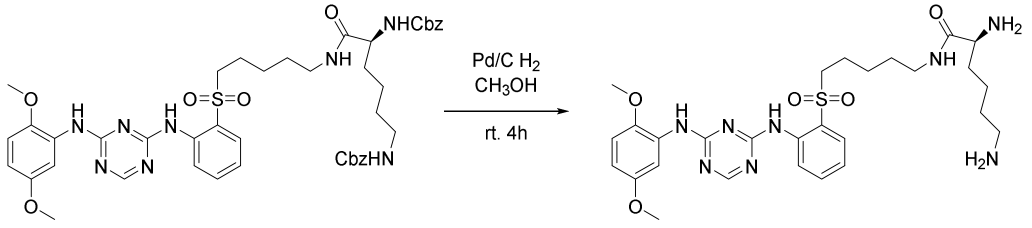

36K5b **36K5**

To a solution of compound **36K5b** (4 mg, 0.005 mmol) in MeOH (2 mL) was added palladium 10% on activated carbon (6 mg). The mixture was stirred under hydrogen atmosphere for 4 hours. The mixture was filtered by celite, washed with MeOH, and concentrated under vacuum. The residue was purified by flash chromatography (DCM/MeOH = 10:1) to give compound **36K5** (2 mg, 72%).

36K5: ^{1}H NMR (600 MHz, CD_3OD) δ 8.40 (s, 1H), 8.37 (s, 1H), 7.93 (dd, *J* = 8.0, 1.5 Hz, 1H), 7.77 (s, 1H), 7.72 (m, 1H), 7.39–7.27 (m, 3H), 6.95 (d, *J* = 8.9 Hz, 1H), 6.66 (dd, *J* = 8.9, 2.9 Hz, 1H), 3.86 (s, 3H), 3.71 (s, 3H), 3.25–3.20 (m, 2H), 3.19–3.17 (m, 1H), 3.06 (m, 2H), 2.65–2.60 (m, 2H), 1.75–1.67 (m, 2H), 1.66–1.63 (m, 2H), 1.52–1.43 (m, 6H), 1.33–1.31 (m, 2H). ^{13}C NMR (151 MHz, CD_3OD) δ 179.0, 176.2, 166.2, 160.1, 153.7, 134.8, 131.0, 129.9, 129.5, 128.8, 128.5, 127.5, 122.3, 117.2, 116.3, 111.1, 69.2, 65.3, 55.5, 53.1, 40.7, 34.8, 31.7, 29.4, 28.1, 25.0, 22.8, 22.3.
HRMS (ESI-TOF) m/z: $[M + Na]^+$ calculated for $C_{28}H_{40}N_8O_5SNa^+$ 623.2735, found 623.2733.
